# Fundamental aspects of long-acting tenofovir alafenamide delivery from subdermal implants for HIV prophylaxis

**DOI:** 10.1038/s41598-022-11020-2

**Published:** 2022-05-17

**Authors:** Manjula Gunawardana, Mariana Remedios-Chan, Debbie Sanchez, Simon Webster, Amalia E. Castonguay, Paul Webster, Christopher Buser, John A. Moss, MyMy Trinh, Martin Beliveau, Craig W. Hendrix, Mark A. Marzinke, Michael Tuck, Richard M. Caprioli, Michelle L. Reyzer, Joseph Kuo, Philippe A. Gallay, Marc M. Baum

**Affiliations:** 1 Department of Chemistry, Oak Crest Institute of Science, 128-132 W. Chestnut Ave., Monrovia, CA USA; 2 Certara Integrated Drug Development, 2000 Peel Street, Suite 570, Montreal, QC Canada; 3 Department of Medicine, Johns Hopkins University, 600 N. Wolfe Street, Baltimore, MD USA; 4 Department of Pathology, Johns Hopkins University, 600 N. Wolfe Street/Carnegie 417, Baltimore, MD USA; 5 Department of Biochemistry, Vanderbilt University, 9160 MRB III, 465 21st Ave. South, Nashville, TN USA; 6 Department of Immunology & Microbiology, The Scripps Research Institute, 10550 North Torrey Pines Road, La Jolla, CA USA

**Keywords:** Drug delivery, Drug development

## Abstract

Global efforts aimed at preventing human immunodeficiency virus type one (HIV-1) infection in vulnerable populations appear to be stalling, limiting our ability to control the epidemic. Long-acting, controlled drug administration from subdermal implants holds significant potential by reducing the compliance burden associated with frequent dosing. We, and others, are exploring the development of complementary subdermal implant technologies delivering the potent prodrug, tenofovir alafenamide (TAF). The current report addresses knowledge gaps in the preclinical pharmacology of long-acting, subdermal TAF delivery using several mouse models. Systemic drug disposition during TAF implant dosing was explained by a multi-compartment pharmacokinetic (PK) model. Imaging mass spectrometry was employed to characterize the spatial distribution of TAF and its principal five metabolites in local tissues surrounding the implant. Humanized mouse studies determined the effective TAF dose for preventing vaginal and rectal HIV-1 acquisition. Our results represent an important step in the development of a safe and effective TAF implant for HIV-1 prevention.

In 2014, UNAIDS launched the “90-90-90: Treatment for All” initiative that, among other aspirational goals, aims to end the AIDS epidemic by 2030^1^. However, in 2019, there were 38.0 million people worldwide living with HIV, 1.7 million new infections, and 690,000 deaths^2^. These alarming statistics suggest success in HIV prevention efforts has stalled, making it increasingly unlikely that the ambitious UNAIDS milestones will be realized. The HIV epidemic is globally heterogeneous, with infection rates in several sub-populations increasing, possibly explaining why the prevention gap has not been reached.

Adolescent girls and young women (ages 15–24) are disproportionately at risk in low-to-middle income countries, with an estimated 7000 new HIV infections occurring weekly^3^. In sub-Saharan Africa, three in four new infections are among girls aged 15–19 years^3^. Exposure to HIV through receptive anal intercourse (RAI) has a higher risk of infection per sex act and, for women, especially those in higher-risk subsets, there is the possibility of both vaginal and rectal exposure during a single sex act^4–6^. In the US and other developed countries, men who have sex with men (MSM) have experienced increases in HIV infection rates and represent the subpopulation most at risk^7^. Highly effective, gender-neutral biomedical modalities for sexual HIV prevention in vulnerable populations are required that can simultaneously provide durable protection in both anatomic compartments^8–12^.

Multiple clinical trials evaluating vaginal and oral antiretroviral (ARV) regimens based on the nucleoside reverse transcriptase inhibitor (NRTI) tenofovir (TFV), alone or in combination with the NRTI emtricitabine (FTC), have shown that HIV pre-exposure prophylaxis (PrEP) can be effective in susceptible men, women, and partners of HIV-infected individuals^13–21^. However, the long-term success of these approaches has been hampered by the compliance burden associated with frequent dosing^22–25^. Long-acting pre-exposure prophylaxis (LA-PrEP) is designed to dramatically reduce dosing frequency, potentially facilitating adherence^26,27^.

A long-acting injectable formulation of the integrase strand transfer inhibitor cabotegravir has demonstrated superiority over daily oral TDF-FTC in the prevention of HIV-1 in cisgender MSM, transgender women, and cisgender women^28,29^. Consequently, an injectable nanoparticle suspension of cabotegravir (200 mg mL^-1^) combined with the non-NRTI rilpivirine (300 mg mL^-1^) became the first long-acting regimen for HIV treatment to receive approval by the US FDA on Jan. 21, 2021^30^. The product is administered once-monthly as two intramuscular continuation injections (2 mL of each formulation) at separate gluteal sites.

Controlled, sustained delivery of the potent prodrug TFV alafenamide (TAF) from a subdermal implant –a LA-PrEP strategy that largely eliminates adherence concerns once the device is placed– has motivated us^31,32^ and others^33–37^ to advance complementary biomedical products with dosing intervals of one year, or longer. Despite these efforts, significant pharmacologic knowledge gaps, and disparate results across research groups, in the preclinical development of TAF subdermal implants remain. The gaps have been exacerbated by the complex, enzyme-driven metabolism of the prodrug as it partitions across anatomic locations to yield TFV diphosphate (TFV-DP), the pharmacologically active moiety against HIV-1, in immune cells that support viral replication. In this work, we rigorously evaluate the pharmacokinetics (PKs) of subdermal TAF delivery in mice and relate these results to drug and drug metabolite distribution in the dermal tissues adjacent to the implants. The implications of our findings in terms of the pharmacodynamic (PD) measures of local safety and efficacy in preventing HIV-1 vaginally and rectally are explored. Our preclinical data suggest that subdermal TAF delivery via implant can safely and predictably prevent sexual HIV-1 infection in mice, warranting further development.

## Results

We have developed a prototype, first-generation implant technology^31,38^ that allows rapid production of devices delivering TAF over a wide range of release rates (*RR*) for preclinical in vivo evaluation^32,38–40^. The implant is a cylindrical silicone tube (dia., 2.5 mm; *L*, 40 mm; *L*, 10 mm for mouse studies) that is perforated with one or more delivery channels (< 1 mm dia.). The tube interior holds a TAF free-base reservoir –compacted powder^31^ or microtablets^38^– containing up to 120 mg TAF in the human-sized devices (*L*, 40 mm). Coating the implant exterior with a controlled release polymer, such as heat-treated poly(vinyl alcohol) (PVA)^31^, provides additional tuning of the in vivo drug delivery kinetics. Drug releases exclusively through the delivery channels, with zero-order kinetics tuned by changing the surface area and number of channels, as well as the sheath coating characteristics.

The results described here relate largely to two mouse-sized (*L*, 10 mm) implant formulations designed for low (target *RR* < 0.3 mg d^-1^) and high (0.3 < target *RR* < 0.75 mg d^-1^) TAF release profiles in C57BL/6 J mice.

### In vivo TAF implant release rates

**Figure 1:**
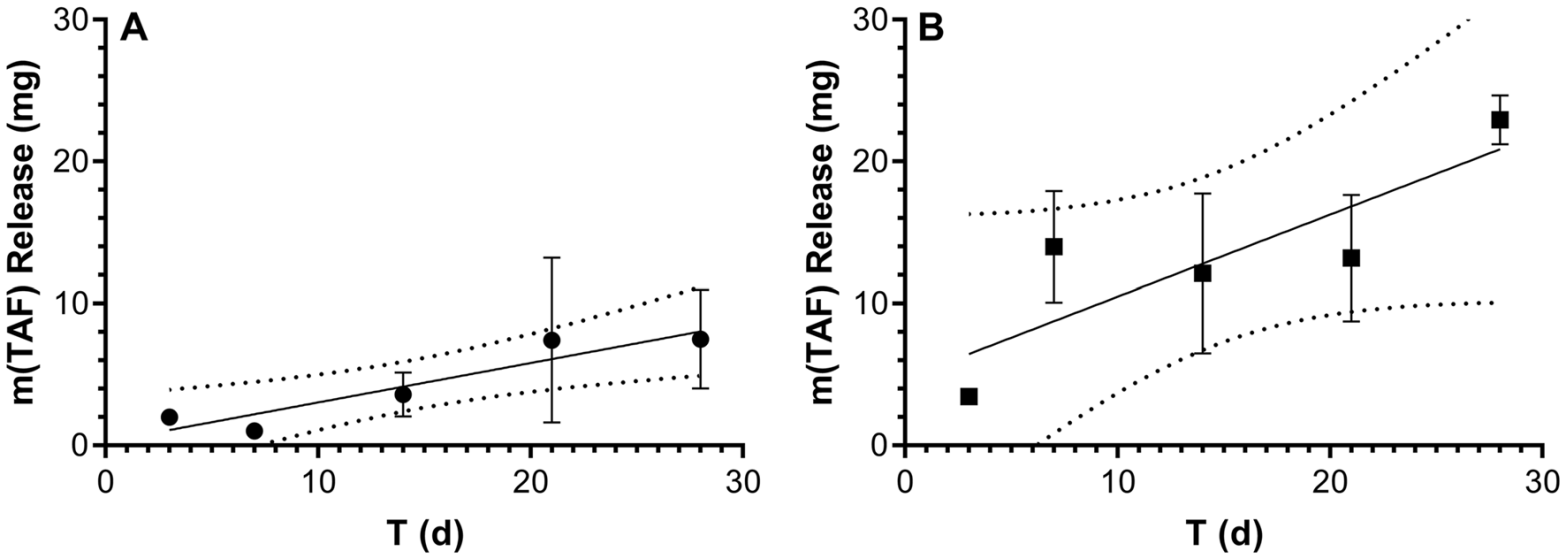
In vivo drug release from TAF implants in C57BL/6 J mice (*N* = 3 per timepoint) estimated via residual drug analysis in used devices. Symbols correspond to means ± SEM; solid line, simple linear regression; broken lines, 95% confidence bands. (**A**) Low-releasing implants; slope = 0.28 ± 0.06 mg d^-1^; *R*^2^ = 0.875. (**B**) High-releasing implants; slope = 0.58 ± 0.21 mg d^-1^; *R*^*2*^ = 0.716.

Cumulative drug release rates from in vivo implant studies typically are calculated from the residual active pharmaceutical ingredient (API) remaining in the device once it is removed at the end of the dosage period. Limited information on the kinetics of in vivo drug release (i.e., linear, first order, or other) is obtained with this approach as only a single timepoint is relied upon. Here, animals were sacrificed at every sample collection timepoint, allowing the in vivo TAF release profiles to be determined empirically for both implants (Fig. 1).

The data shown in Fig. 1 suggest that implant release was linear over the 28-day dosing period, as predicted based on in vitro TAF release kinetics^31,32^. While some expected variability was observed, the quality of the linear regressions (Fig. 1A *R*^2^ = 0.875; Fig. 1B, *R*^2^ = 0.716) is acceptable, considering that each symbol in Fig. 1 represents means from three implants in three separate animals. The mass of TAF per implant in these groups prior to placement was 24.8 ± 0.91 mg (mean ± *SD*). The mean TAF mass remaining in the used implants retrieved on Day 28 was: low-releasing, 19.9 mg; high-releasing, 2.2 mg.

**Scheme 1:**
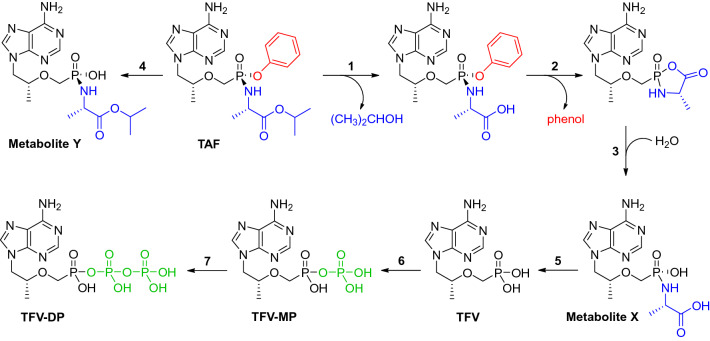
The TAF metabolism is complex and includes five principal, isolable metabolites and multiple enzyme-driven steps ^43,51–53^.

In vivo TAF degradation (see Scheme 1) in the implants during use is a potential concern with prolonged exposure to biological fluids of a depot containing the chemically labile prodrug. We measured the composition of the compound(s) remaining in retrieved implants by HPLC to determine if in vivo TAF degradation had occurred. The peak areas corresponding to TAF typically made up > 98% of the total peak area (TAF and decomposition products) in the chromatograms, irrespective of the implant group. The main observed impurity was TFV, also present at a similar concentration in the drug substance (purity *ca*. 98%) used to formulate the implants. Generally, less than 1% of the impurities were formed during in vivo implant use. TAF remained as a solid (in this case compacted as microtablets, vide infra) until it was dissolved by subcutaneous fluids entering through the delivery channel(s), likely contributing to the high observed in vivo stability.

### TAF Pharmacokinetics following intravenous and subcutaneous injection

To enable PK modeling of systemic TAF and its metabolites, we conducted the necessary foundational studies in C57BL/6 J mice following intravenous (IV) and subcutaneous (SQ) bolus TAF dosing, as these data were not available in the literature.

**Figure 2:**
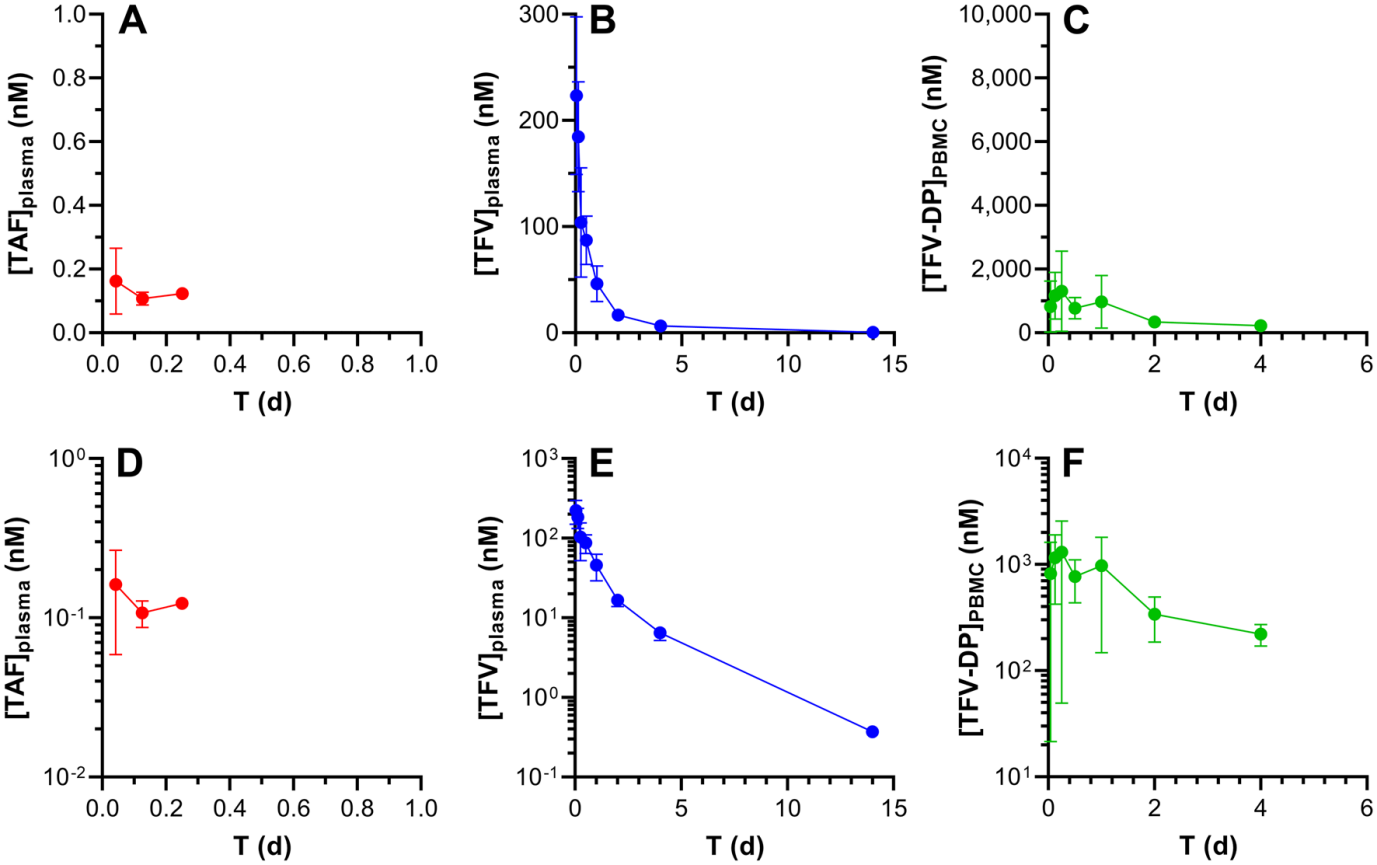
Intravenous TAF injection in C57BL/6 J mice results in different pharmacokinetics across the three key analytes. The data are shown as linear (**A**–**C**) and semilog (**D**–**F**) plots and all concentration units are represented in nanomolar (nM) for ease of comparison. Concentration–time profiles of plasma TAF (**A** and **D**; red circles), plasma TFV (**B** and **E**; blue circles), and PMBC TFV-DP (**C** and **F**; green circles) are shown as means ± SD (*N* = 4 per group). The assay lower limits of quantitation (LLQs) are: TAF, 0.06 nM (0.03 ng mL^-1^); TFV, 3.5 nM (1 ng mL^-1^); TFV-DP, 18 nM (based on 5 fmol/sample, and a median of 1.4 × 10^6^ PBMCs per sample).

**Figure 3:**
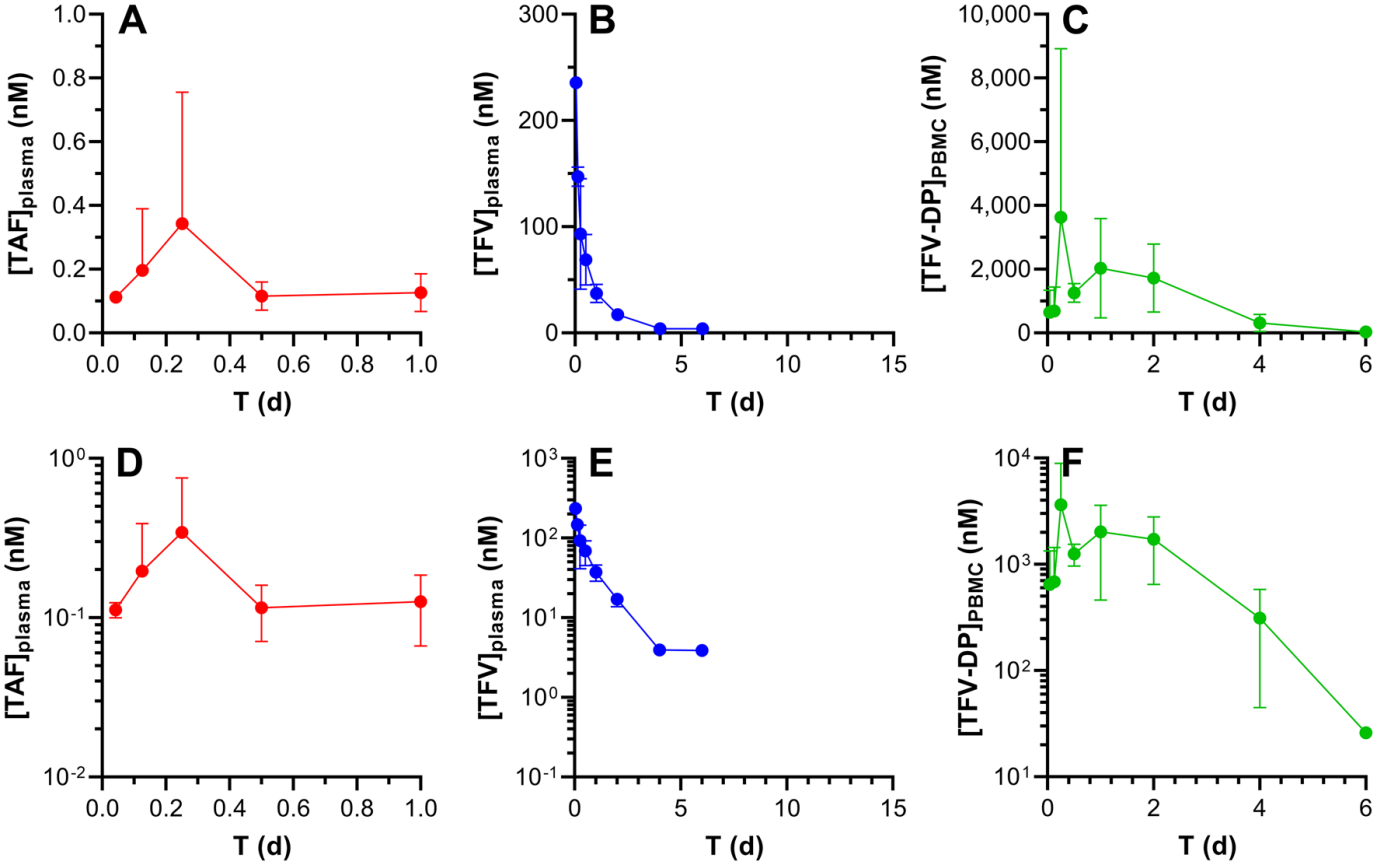
Subcutaneous TAF injection in C57BL/6 J mice results in different pharmacokinetics across the three key analytes. The data are shown as linear (**A**–**C**) and semilog (**D**–**F**) plots and all concentration units are represented in nanomolar (nM) for ease of comparison. Concentration–time profiles of plasma TAF (**A** and **D**; red circles), plasma TFV (**B** and **E**; blue circles), and PMBC TFV-DP (**C** and **F**; green circles) are shown as means ± SD (*N* = 4 per group). The assay LLOQs are: TAF, 0.06 nM (0.03 ng mL^-1^); TFV, 3.5 nM (1 ng mL^-1^); TFV-DP, 5 nM (based on 5 fmol/sample, and a median of 5.0 × 10^6^ PBMCs per sample).

Plasma TAF and tenofovir (TFV) plasma as well as peripheral blood mononuclear cell (PBMC) TFV-DP concentration–time profiles are similar when TAF is administered by IV (Fig. 2) or SQ (Fig. 3) injection. It should be noted that TAF is highly unstable in mouse plasma, and rapidly converts to TFV^41^, partially explaining why the prodrug only was transiently quantifiable at low concentrations in early timepoints. All concentrations were normalized to nanomolar (nM) for ease of comparison across the datasets. Intracellular TFV-DP concentrations were calculated assuming a mean cell volume of 0.2 µL/10^6^ PBMCs^42^. The following conversions from commonly reported units are included for the sake of convenience: plasma TAF, 1.0 ng mL^-1^ = 2.1 nM; plasma TFV, 1.0 ng mL^-1^ = 3.5 nM; PBMC TFV-DP, 1.0 fmol/10^6^ cells = 5.0 nM. Both linear (Figs. 2, 3A–C) and semilog (Figs. 2, 3D–F) plots are presented to allow complete visualization of the concentration–time profiles.

**Figure 4:**
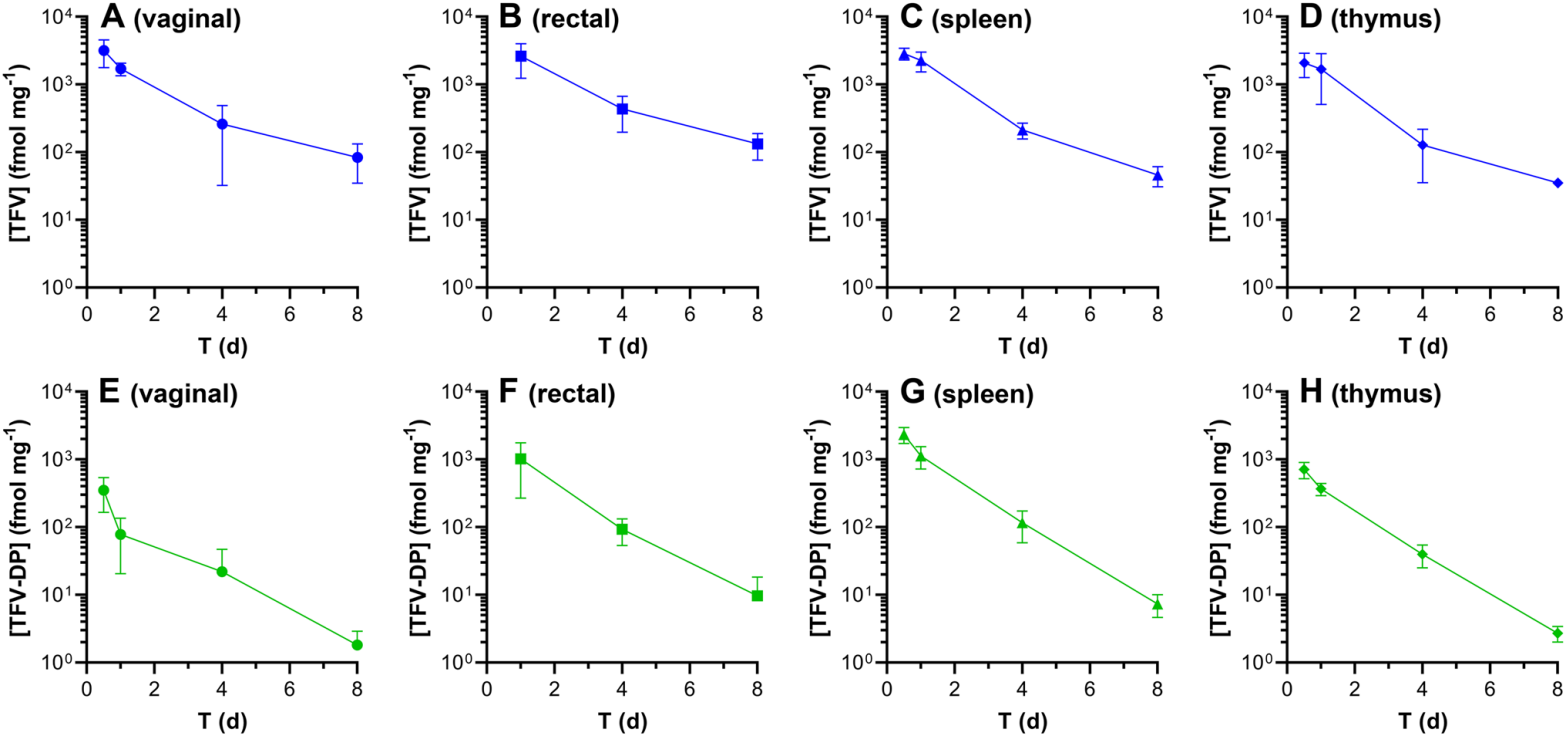
Tissue homogenate drug concentration–time profiles following intravenous TAF dosing in C57BL/6 J mice. Blue, TFV; green, TFV-DP. The sampled anatomic compartments are: (**A**) and (**E**), vaginal (median tissue mass, 192 mg/sample); (**B**) and (**F**) rectal (median tissue mass, 71 mg/sample); (**C**) and (**G**) spleen (median tissue mass, 106 mg/sample); and (**D**) and (**H**) thymus (median tissue mass, 68 mg/sample). The assay LLOQs are: TFV (based on 0.05 ng/sample), vaginal 0.9 fmol mg^-1^; rectal, 2.4 fmol mg^-1^; spleen, 1.6 fmol mg^-1^; thymus, 2.6 fmol mg^-1^; TFV-DP (based on 5 fmol/sample), vaginal 0.03 fmol mg^-1^; rectal, 0.07 fmol mg^-1^; spleen, 0.05 fmol mg^-1^; thymus, 0.07 fmol mg^-1^.

Tissue homogenate TFV and TFV-DP concentrations (both in fmol mg^-1^) as a function of time in key anatomic locations for HIV-1 PrEP following TAF IV dosing are shown in Fig. 4. The prolonged (> 8 days) TFV-DP concentrations in these tissues following a single bolus TAF administration is an important confirmation of the prodrug’s ability to efficiently distribute into immune cells in vivo, where it is metabolized to TFV-DP^43^. Terminal half-lives, expressed as mean ± standard deviation, of TAF metabolites in tissue samples were estimated by noncompartmental analysis (NCA) using the data shown in Fig. 4: TFV: vaginal, 1.63 ± 0.35 d; rectal, 1.79 ± 0.57 d; spleen, 1.27 ± 0.032 d; thymus, 0.839 ± 0.26; TFV-DP: vaginal, 1.30 ± 0.15 d; rectal, 1.10 ± 0.33 d; spleen, 0.969 ± 0.041 d; thymus, 0.971 ± 0.025.

### Subdermal TAF implant pharmacokinetics

Having established the PK baseline using IV and SQ single (bolus) TAF injections, we investigated the drug metabolite concentration–time profiles during 28 days of implant use in C57BL/6 J mice. Both implant formulations (low- and high-releasing) were evaluated in separate arms.

**Figure 5:**
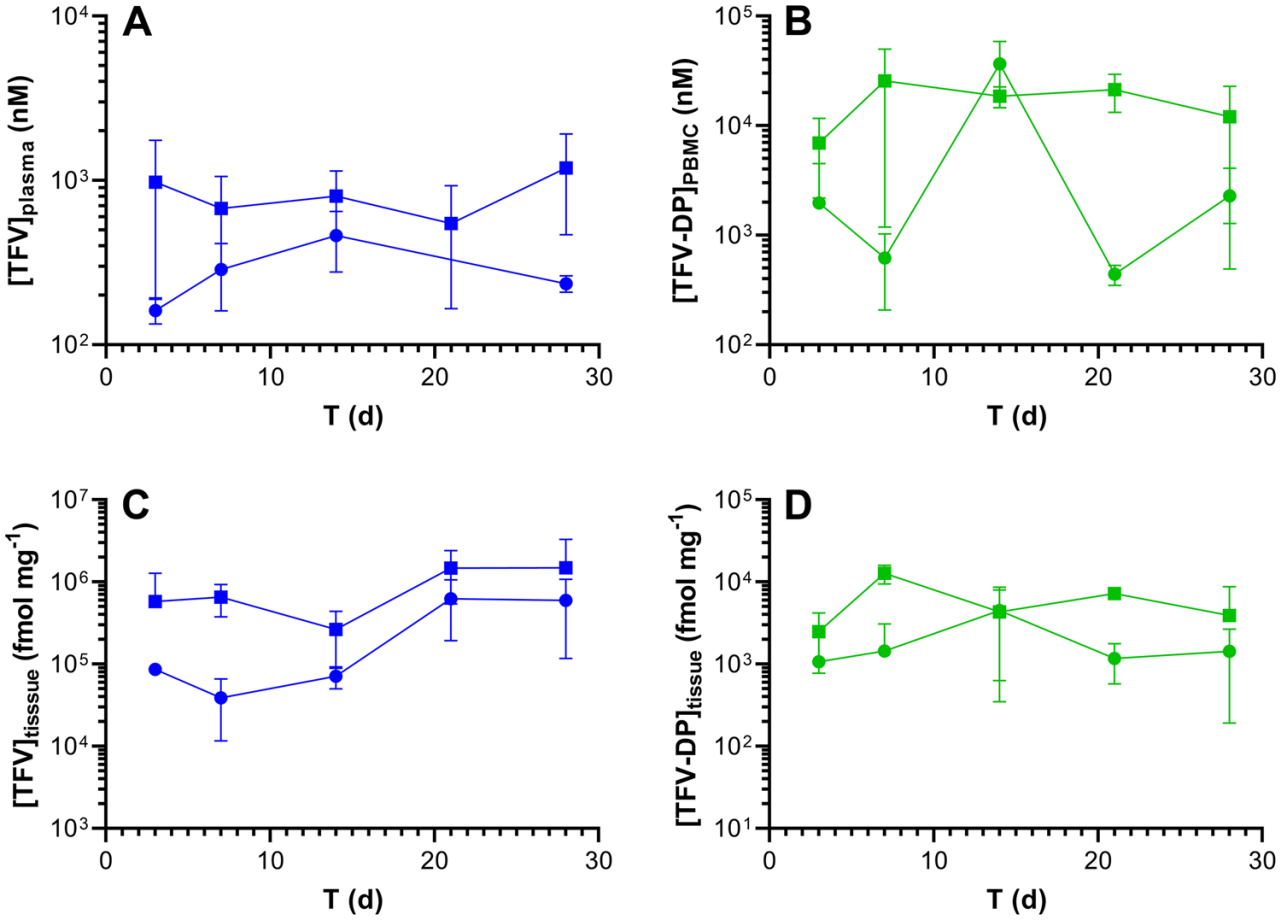
Subdermal placement of TAF implants in C57BL/6 J mice maintains sustained drug concentrations. Data are presented as means ± *SD* (*N* = 3 per timepoint); circles, low-releasing implants (0.28 mg d^-1^ in vivo); squares, high-releasing implants (0.58 mg d^-1^ in vivo). Pharmacokinetic profiles of (**A**) plasma TFV; (**B**) PBMC TFV-DP; (**C**) dermal tissue (collected adjacent to the implant) TFV; and (**D**) dermal tissue TFV-DP. The assay LLOQs are: plasma TFV, 3.5 nM (1 ng mL^-1^); PBMC TFV-DP, 13 nM (based on 5 fmol/sample, and a median of 1.9 × 10^6^ PBMCs per sample); tissue TFV 21 fmol mg^-1^ (based on 0.05 ng/sample, and a median of 8.4 mg per sample); tissue TFV-DP 0.6 fmol mg^-1^ (based on 5 fmol/sample, and a median of 8.4 mg per sample).

TFV plasma and TFV-DP PBMC concentration *versus* time as well as TFV and TFV-DP dermal tissue (collected adjacent to the implant) concentration *versus* time plots comparing both implant groups are presented in Fig. 5 and summarized in Table 1.

**Table 1:** Summary of equilibrium (Day 3–28) TFV and TFV-DP concentrations during implant use.

Implant, analyte, matrix^a^ units	*n*	% Quantifiable	Median (IQR)^b^
Low, TFV, plasma nM ng mL^-1^	10	70^c^	183.5 (12.4–253.1) 52.7 (4.2–72.7)
Low, TFV-DP, PBMC nM fmol/10^6^ cells	15	100	772.9 (387.8–4,306) 154.6 (77.6–861.1)
Low, TFV, dermal tissue fmol mg^-1^ ng mg^-1^	15	100	82.6 × 10^3^ (57.0 × 10^3^–415.0 × 10^3^) 23.7 (16.4–119.2)
Low, TFV-DP, dermal tissue fmol mg^-1^	15	100	1.448 (518.6–2.429)
High, TFV, plasma nM ng mL^-1^	15	100	1.070 (673.5–1.695) 307.4 (193.5–486.8)
High, TFV-DP, PBMC nM fmol/10^6^ cells	15	100	14,987 (8.784–21,531) 2.997 (1.757–4.306)
High, TFV, dermal tissue fmol mg^-1^ ng mg^-1^	15	100	540.1 × 10^3^ (292.1 × 10^3^–1.156 × 10^3^) 155.1 (83.9–332.1)
High, TFV-DP, dermal tissue fmol mg^-1^	15	100	6.303 (2.351–8.846)

Low, 0.28 mg d^-1^ TAF release in vivo; high, 0.58 mg d^-1^ TAF release in vivo.

^a^All values correspond to time points (D3-28) with the implant in place.

^b^Interquartile range, between first (25th percentile) and third (75th percentile) quartiles.

^c^BLQ values were omitted from the analysis.

We do not have a good explanation for the high TFV-DP PBMC concentrations observed for the low-releasing implant on Day 14 (Fig. 5B). The mean concentration is not biased high by an outlier, as evidenced by the low standard deviation. In addition, the three implants from the low-releasing group did not have an uncharacteristically high in vivo release rate on Day 14, as supported by Fig. 1A.

Median, equilibrium plasma TFV concentrations in the high-releasing implant group were 5.8 × those in the low releasing group, while the corresponding PBMC TFV-DP concentrations were 19.4 × higher. Median, equilibrium, local dermal tissue TFV concentrations were 6.5 × higher in the high-releasing implant group when compared to the low-releasing group, and the corresponding median TFV-DP concentrations were 4.4 × higher.

### Tissue TFV-DP mole fraction

**Figure 6:**
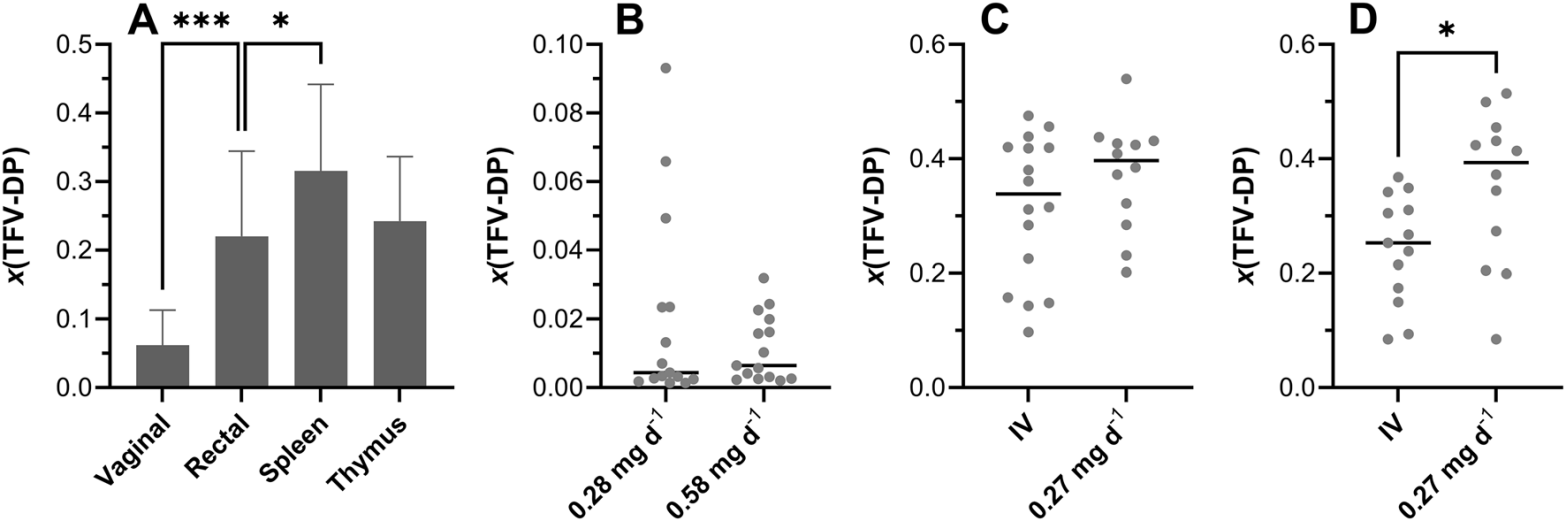
Tissue homogenate TFV-DP mole fractions, *x*(TFV-DP), and mole fraction distributions are different across anatomic compartments following TAF administration (IV and implant) in C57BL/6 J mice. (**A**) IV TAF dosing study; a Kruskal–Wallis test (unpaired, nonparametric) was used to compare all four groups and found that the *x*(TFV-DP) values were significantly different (*P* < 0.0001). The vaginal *versus* rectal (*P* = 0.0001, ***) TFV-DP mole fractions and the spleen *versus* rectal (*P* = 0.0468, *) TFV-DP mole fractions were found to be significantly different using a Mann–Whitney *t*-test (unpaired, nonparametric). The thymus *versus* spleen (*P* = 0.0755) and thymus *versus* rectal (*P* = 0.6501) TFV-DP mole fractions were found to be not significantly different using the same *t*-test. (**B**) Scatter plot showing distributions of dermal *x*(TFV-DP) values for low- (0.28 mg d^-1^ in vivo) and high- (0.58 mg d^-1^ in vivo) releasing TAF implant groups; circles represent individual measurements/animals and horizontal lines represent medians. The data were not found to be significantly different (*P* = 0.9349) using a Mann–Whitney *t*-test. (**C**) Scatter plot showing distributions of spleen *x*(TFV-DP) values for IV dosing and low- (0.27 mg d^-1^ in vivo) releasing TAF implant groups; circles represent individual measurements/animals and horizontal lines represent medians. The data were not found to be significantly different (*P* = 0.2803) using a Mann–Whitney *t*-test. (**D**) Scatter plot showing distributions of thymus *x*(TFV-DP) values for IV dosing and low- (0.27 mg d^-1^ in vivo) releasing TAF implant groups; circles represent individual measurements/animals and horizontal lines represent medians. The data were found to be significantly different (*P* = 0.0257) using a Mann–Whitney *t*-test.

The TFV-DP mole fraction, *x*(TFV-DP) [i.e., (number of moles of TFV-DP)/(number of moles of TFV + number of moles of TFV-DP)] on a per sample basis, in tissue homogenate from various anatomic locations is compared across TAF dosing modalities in Fig. 6.

The distribution of *x*(TFV-DP) values in tissue homogenate was variable across anatomic compartments (Fig. 6A). The median (IQR) vaginal tissue *x*(TFV-DP) was 0.051 (0.022–0.080), indicating that 5.1% of the total TFV was present as TFV-DP and was significantly lower than in the other tissue types. Median (IQR) *x*(TFV-DP) values in rectal, spleen, and thymus samples were 0.24 (0.099–0.30), 0.34 (0.21–0.42), and 0.25 (0.17–0.31), respectively. Dermal tissues adjacent to TAF implants had the lowest median (IQR) *x*(TFV-DP) values: low-releasing, 0.0044 (0.0026–0.024); high releasing, 0.0065 (0.0029–0.018); i.e., TFV-DP made up less than 1% of the total TFV species. There was no significant difference between the dermal tissue *x*(TFV-DP) values in the low- and high-releasing implant groups (Fig. 6B) using a Mann–Whitney *t*-test (*P* = 0.9349).

Spleen and thymus samples were collected in a separate implant study, where the in vivo TAF release rate was 0.27 mg d^-1^, similar to the low-releasing implants. In this study, there was no significant difference between the *x*(TFV-DP) values when comparing spleen tissue homogenate from the implant group to the IV group (Fig. 6C) using a Mann–Whitney *t*-test (*P* = 0.2803), but the corresponding thymus *x*(TFV-DP) values (Fig. 6D) were significantly different (*P* = 0.0257) using the same test.

### TAF Bioavailability following IV and SQ injection

The plasma TFV concentration–time profiles for the IV (Fig. 2B and E) and SQ (Fig. 3B and E) groups as well as the PBMC TFV-DP concentration–time profiles for the IV (Fig. 2C and F) and SQ (Fig. 3C and F) groups indicate a lack of flip-flop kinetics, evidenced by parallel terminal (i.e., elimination) phases over the final timepoints. Generally, these mono-exponential declining phases suggest that analyte elimination is truly related to the elimination from the body rather than absorption from the SQ dosing site. Had flip-flop kinetics been demonstrated with SQ dosing (via injection), due to an absorption rate from the SQ compartment far slower than elimination from the central compartment, it would have resulted in much slower, or shallower, slopes in the SQ profile compared to the corresponding IV profile. True and robust elimination half-lives (*t*_1/2_) therefore could be estimated (Table 2).

**Table 2:** Model describing the systemic PK parameters for TFV, using IV and SQ co-modeling.

Parameter	Units	Estimate	CV (%)
*V*	L	4.59	10.8
*CL*	L d^-1^	6.45	6.3
*V*_p_	L	6.32	14.2
*CL*_p_	L d^-1^	2.94	26.0
*t*_1/2_	d	2.3	NA
*F*		0.97	12.3
*K*_out_	d^-1^	2.33	26.9
*V*_m_/*K*_m_	L d^-1^	1.15 × 10^–5^	23.4
**Model error**			
Plasma	%	34.4	14.7
PBMC	%	82.3	10.0

*V*, Volume of distribution of the central compartment; *CL*, Total body clearance; *V*_p_, Volume of distribution of the second compartment; *CL*_*p*_, Clearance from the second compartment; *t*_1/2_, Elimination half-life; *F*, Bioavailability/exposure scaling; *K*_a_, Absorption rate parameter; *V*_cell_, PBMC physiological volume; *K*_out_, Elimination rate parameter; *V*_m_, Maximum rate achieved by the system using Michaelis–Menten kinetics; *K*_m_, Michaelis constant; multiplicative error, error that is proportional to concentration.

Comparing the drug and drug metabolite PK following SQ TAF injection to the corresponding PK in the IV TAF dosing groups allowed SQ bioavailabilities (*F*) to be calculated: (a) using plasma TFV concentration data only, in an independent analysis based on areas-under-the-curve (AUCs), *F* = 87%; and (b) using plasma TFV and PBMC TFV-DP concentration data in a parameterized model, *F* = 97%. In both cases, *F* was close to 100% and observed deviations were within the inherent errors of the experiment (e.g., dosing, sample collection/processing, bioanalytical methods). Based on these results, it was assumed that the drug was completely absorbed following SQ administration.

The rapid in vivo TAF metabolism to multiple compounds has led us to calculate *F* for the prodrug in terms of TFV and TFV-DP systemic or intracellular exposure (i.e., dose-normalized exposure AUC). To avoid confusion that may arise from the use of the term “bioavailability”, we will use “exposure scaling” to describe the extent (expressed as %) TFV (plasma) and TFV-DP (PBMC) reach their target compartment relative to IV (and SQ) dosing via injection.

### Modeling and simulation TAF implant PK based on TAF IV and SQ data

**Figure 7:**
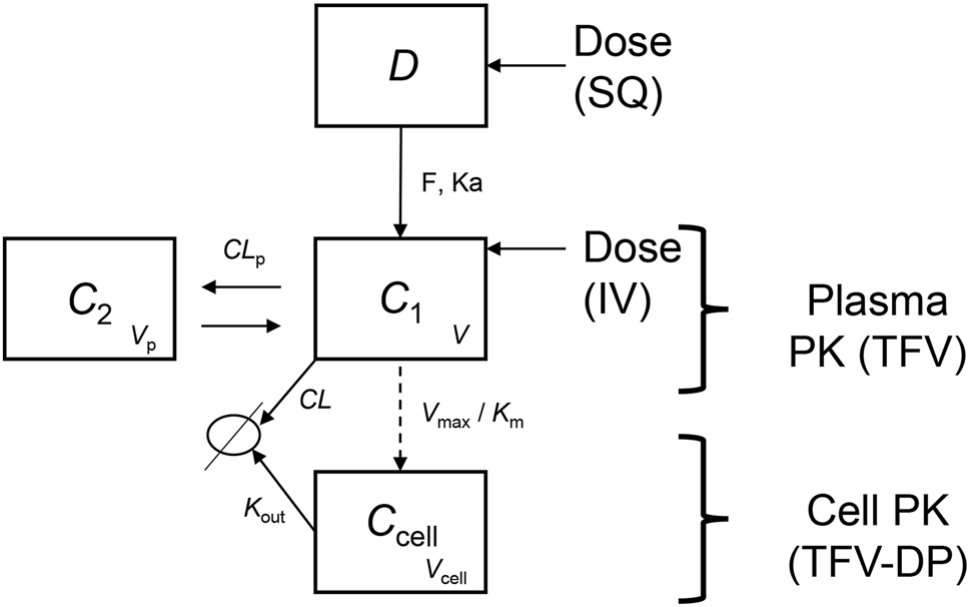
Pharmacokinetic model for plasma TFV and intracellular TFV-DP in mice, based on the model reported by Duwal et al.^44^. IV dosing occurs in the *C1* compartment, SQ and implant dosing occur in the *D* compartment (dosing reservoir). The release rate into the *D* compartment is assumed instantaneous following SQ dosing, but is governed by the actual implant TAF release rate in the case of implant administration. The fraction of TAF metabolized to TFV is assumed to be 100%. *V*, volume of distribution of the central compartment; *CL*, total body clearance; *V*_p_, volume of distribution of the second compartment; *CL*_p_, clearance from the second compartment; *t*_1/2_, elimination half-life; *F*, bioavailability/exposure scaling; *K*_a_, absorption rate parameter; *V*_cell_, PBMC physiological volume; *K*_out_, elimination rate parameter; *V*_m_, maximum rate achieved by the system using Michaelis–Menten kinetics; *K*_m_, Michaelis constant.

A literature PK model^44^ was adapted (Fig. 7) and used to estimate TAF systemic PK parameters in mice by simultaneously modeling measured TFV and TFV-DP concentrations following IV and SQ TAF injection. The results of the simultaneous model (co-model) are summarized in Table 2, and the parameters were used in subsequent simulation exercises (vide infra).

The model described in Fig. 7 was transcribed in a series of differential equations (vide infra) within the WinNonLin software and applied to the TAF injection SQ and IV mouse data. The change in amount over time within each compartment was related to the difference in the input into the compartment (e.g., the Dose × *F* × *K*_a_ term into compartment *C*_1_) and the output from the compartment (e.g., the Concentration × *K*_out_ term for compartment *C*_cell_). Two compartments –plus a dosing compartment– best described TFV plasma PKs.

A third compartment was used to model the PKs of PBMC TFV-DP concentrations. Intracellular PKs of TFV-DP were linked to the plasma TFV concentration via saturable uptake (*V*_max_ and *K*_m_) with maximum velocity of uptake and first order elimination kinetics, *K*_out_. The TFV/TFV-DP PK model therefore is made up of four compartments (Fig. 7): “Dose” represents the mass of TFV in the dosing reservoir, based on a molar equivalent from the TAF drug content and the valid assumption that TAF is converted rapidly and completely to TFV in vivo. *C*_1_ is the central compartment, representing the TFV plasma concentration in the body. The second compartment *C*_2_ represents the peripheral tissues, and the cellular compartment *C*_cell_ represents the concentrations of TFV-DP in PBMCs. Parameters *CL*_p_ and *V*_p_ describe clearance and volume of the peripheral compartment (*C*_2_) and *K*_a_ and *CL* are the rates of TFV uptake and clearance into/out of the central compartment (*C*_1_), respectively. The parameter *K*_a_ was fixed in Duwal et al.^44^ and also was fixed here to 8 h^-1^, due to the lack of clear data in the absorption phase. The physiological PBMC volume was assumed to be 7.55 × 10^–7^ L/10^6^ cells, to remain consistent with Duwal et al.^44^ for the model analysis, while all other parameters were estimated in the model. The ordinary differential equations (Eqs. 1–3) used in the final model are presented below:

1 $$\frac{d}{dt}C_{1} \left( t \right) = \frac{{F \cdot K_{{\text{a}}} \cdot D\left( t \right)}}{V} - C_{1} \left( t \right) \cdot \frac{CL}{V} - \frac{{CL_{{\text{p}}} }}{V} \cdot C_{1} \left( t \right) + \frac{{CL_{{\text{p}}} }}{{V_{{\text{p}}} }} \cdot C_{2} \left( t \right)$$

2 $$\frac{d}{dt}C_{2} \left( t \right) = \frac{CL}{V} \cdot C_{1} \left( t \right) + \frac{{CL_{{\text{p}}} }}{{V_{{\text{p}}} }} \cdot C_{2} \left( t \right)$$

3 $$\frac{d}{dt}C_{{{\text{cell}}}} \left( t \right) = \frac{{V_{{{\text{max}}}} \cdot C_{1} \left( t \right)}}{{K_{{\text{m}}} }} - C_{{{\text{cell}}}} \left( t \right) \cdot K_{{{\text{out}}}}$$

No improvements in goodness-of-fit were observed in preliminary model runs when the TAF bioavailability was varied over the 87–120% range. There also were no improvements in the modeled data quality when an apparent rapid mean absorption time was estimated as an input rate from the subcutaneous depot (i.e., peak values occurred at the first timepoint post dose, or 1 h).

The elimination half-life, *t*_1/2_, for TFV in plasma was 53 h (2.2 d, Table 2), and the contribution of the non-linear Michaelis–Menten intrinsic clearance was found to be negligible: *V*_max_/*K*_m_ = 0.1 L d^-1^, or 2% of the non-saturable clearance. Reports on plasma TFV PK following parenteral TAF dosing in mice are limited. Prathipati et al. studied the relative availability of TFV following subcutaneous TAF-loaded nanoparticles or free drug in humanized mice^45^. The plasma TFV *t*_1/2_ was found to be 14 h following subcutaneous dosing of the free drug, significantly lower than the 53 h measured here. Furthermore, there was no evidence of multicompartment kinetics, as reflected by the developed one-compartment PK model for systemic TFV. Our results suggest that TFV displays multi-exponential kinetics in mice.

For TFV-DP in PBMCs, *t*_1/2_ was dependent on the dosing route. For IV and SQ TAF injection, *t*_1/2_ was found to be 41 and 17 h, respectively (see “Discussion” for possible explanations). This value in mice is somewhat consistent (IV dosing) with reports in humans where the intracellular (PBMC) TFV-DP half-life of healthy individuals was estimated as 48 h^46^.

### Simulation of plasma TFV and PBMC TFV-DP concentrations following subdermal TAF delivery from implants

**Figure 8:**
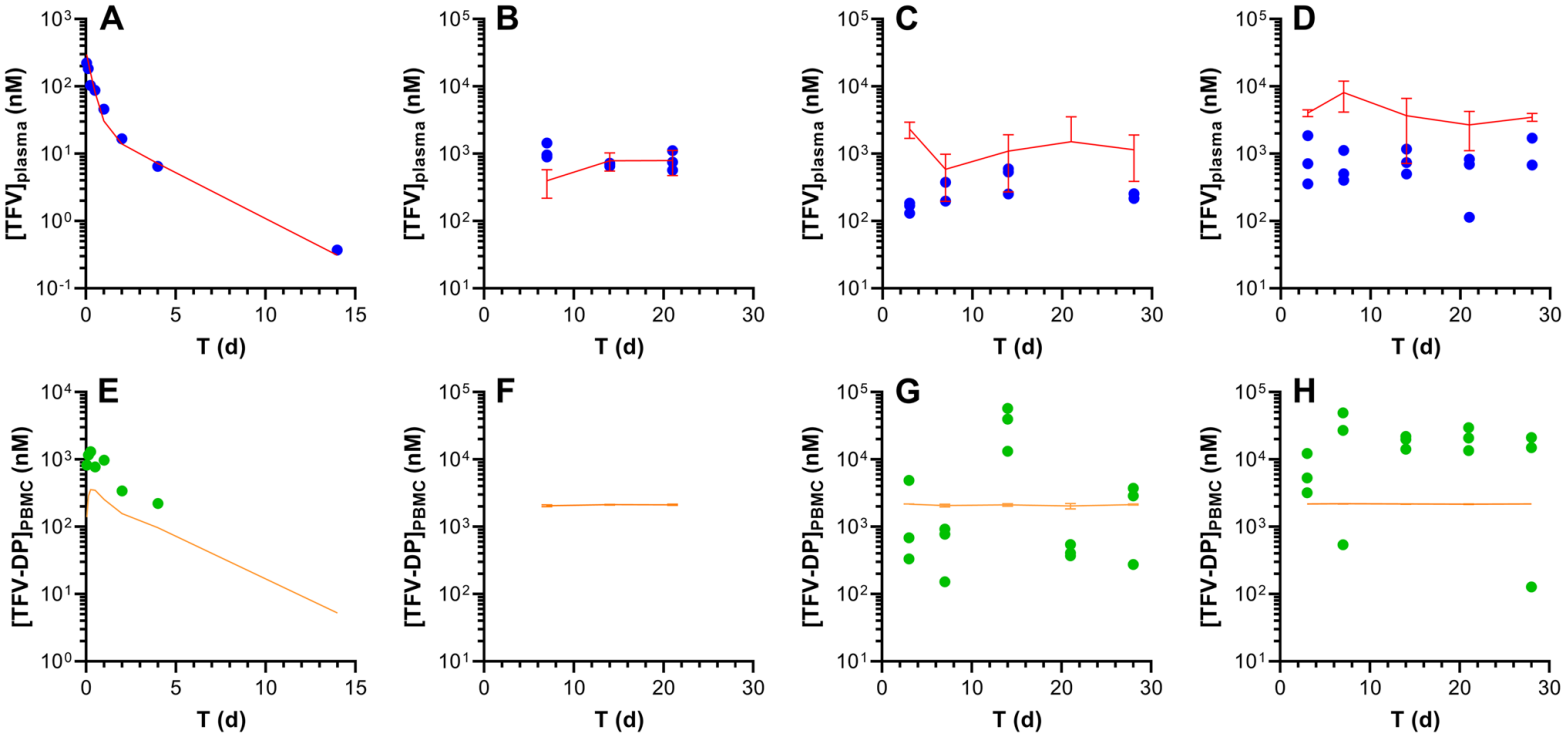
Simulation of plasma TFV and intracellular (PBMC) TFV-DP concentration–time profiles in mice following TAF dosing, using the IV/SQ PK model (Fig. 7) and implant release characteristics (Fig. 1). Circles represent individual experimental data; blue, plasma TFV; green, PBMC TFV-DP; lines represent model simulations (mean ± *SD*); red, plasma TFV; orange, PBMC TFV-DP. (**A** and **E**) goodness-of-fit of IV data used an internal validation. (**B** and **F**) Simulation of plasma TFV and PBMC TFV-DP concentrations for a low-releasing (0.23 mg d^-1^) implant from a previous study^32^, where PBMC TFV-DP concentrations were not measured. (**C** and **G**) Simulation of plasma TFV and PBMC TFV-DP concentrations for the low-releasing (0.28 mg d^-1^) implant group. (**D** and **H**) Simulation of plasma TFV and PBMC TFV-DP concentrations for the high-releasing (0.58 mg d^-1^) implant group.

The PK model (Fig. 7) and parameters (Table 2) calculated based on the drug-concentration profiles in plasma and PBMCs following IV and SQ injections were employed to simulate systemic drug exposure following TAF delivery from subcutaneous implants, utilizing only the device in vivo release rates as inputs. The results are summarized in Fig. 8.

The measured in vivo implant TAF release rates were used to derive the concentrations in the absorption compartment (*C*_1_). All other parameters were unmodified from Table 2. Our starting hypothesis was that the difference between SQ injection and long-acting SQ delivery via implant would be strictly related to the implant release rate. Once the drug was released from the device, it was hypothesized to be transferred from the SQ pocket to body circulation via the same mechanism and governed by the same parameters as by SQ injection, and therefore the same model was employed. In reviewing the initial modeling results, using a TAF implant exposure scaling of 100% relative to SQ (and IV) administration via injection could not accurately predict the implant plasma TFV concentration–time data. Good fit results between the predicted and observed data were obtained by increasing the exposure scaling to 400%, relative to SQ TAF injection (Fig. 8). Exposure scaling (i.e., apparent bioavailability) can only be reliably estimated when the following assumptions are met: (a) clearances do not change between routes; and (b) elimination/absorption follow first order kinetics. Following insertion of the implant, apparent clearance (i.e., *CL*/*F*) was decreased fourfold to 1.48 L d^-1^ possibly indicating that the elimination was governed by the implant release rate rather than representing true systemic elimination (i.e., flip-flop kinetics; note that while flip-flop kinetics were not observed following bolus dosing, they cannot be ruled out for implant, long-acting, dosing), or that absorption from the SQ pocket was non-linear, a highly unexpected result as discussed further below.

Overall, following the above adjustments, there was agreement between model simulations and observed data (Fig. 8), using only implant release characteristics and disposition kinetics from the IV/SQ injection model (Fig. 7). Intracellular (PBMC) TFV-DP concentrations in the high-releasing (0.58 mg d^-1^) implant group were underestimated by the model. Additional simulations suggested that a rate constant for PBMC loading for this implant needed to be increased by fivefold (i.e., exposure scaling of 500%) for the model to predict the observed TFV-DP concentrations.

### Drug and drug metabolite distribution imaging in dermal tissues adjacent to the implant

Matrix-assisted laser desorption/ionization (MALDI) imaging mass spectrometry (IMS) allows in situ mapping of a wide range of analytes –including small molecule drugs, metabolites, peptides, lipids, and proteins– within a thin tissue section^47–50^. A focused laser beam directly desorbs and ionizes molecules from predetermined, discrete locations in the tissue, and the resulting ions subsequently are analyzed by mass spectrometry, generating a two-dimensional distribution profile for each ion fragment pair (analyte). The technique provides spatial and molecular specificity by measuring a unique mass-to-charge ratio (*m/z*), corresponding to the target analyte, and concomitant ion fragmentation at specific coordinates in the tissue section. The precise and sensitive detection method affords greater specificity (i.e., higher confidence of correctly identifying the analyte) than other imaging approaches such as fluorescence microscopy and autoradiography.

**Figure 9:**
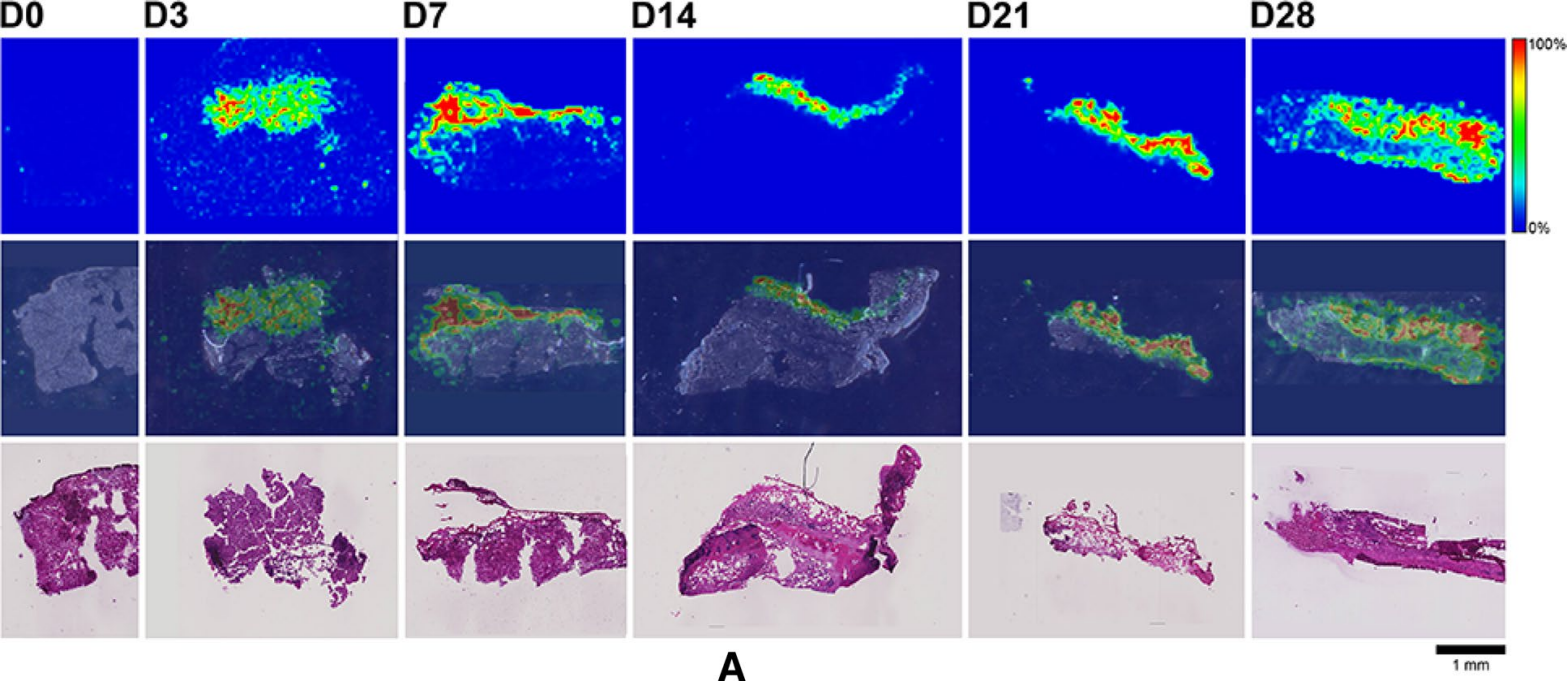

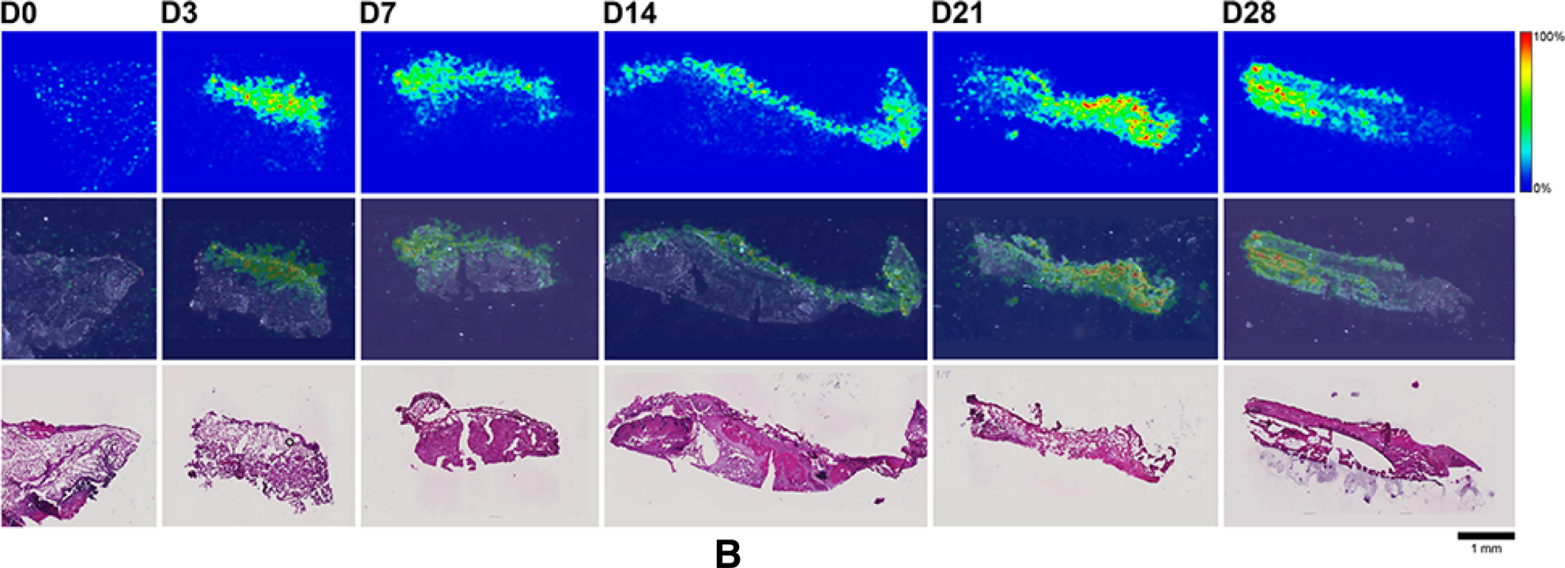

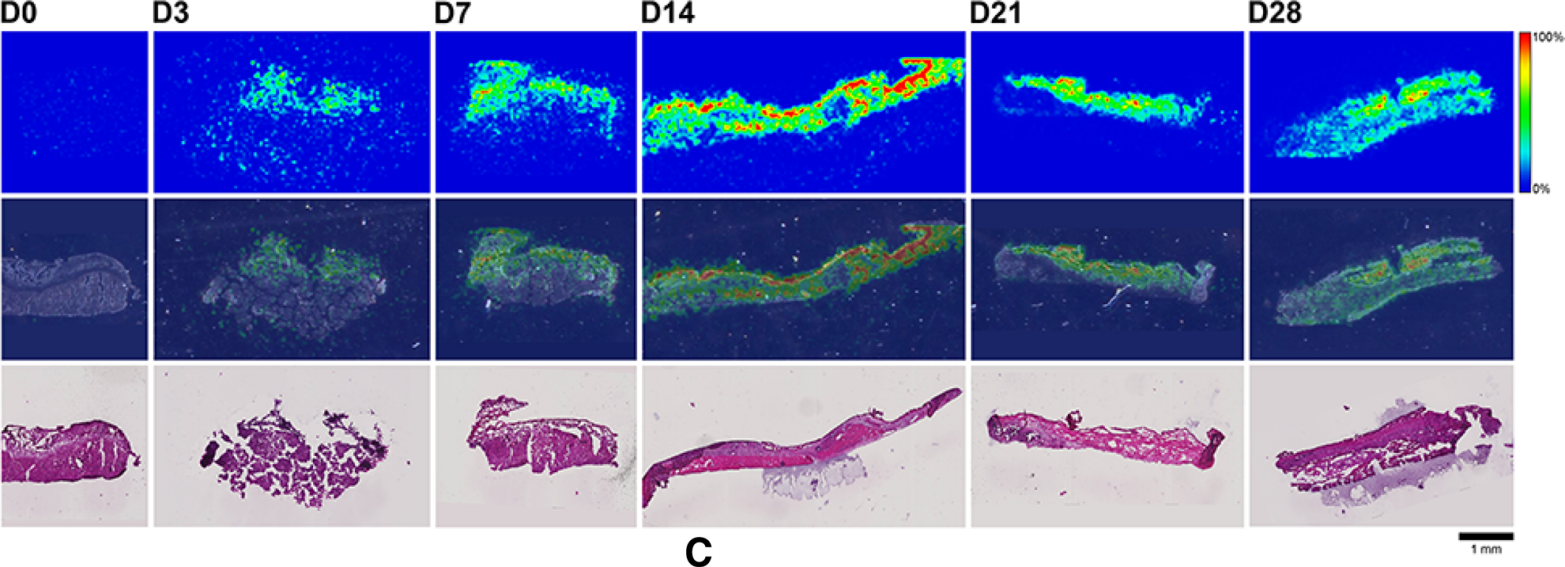

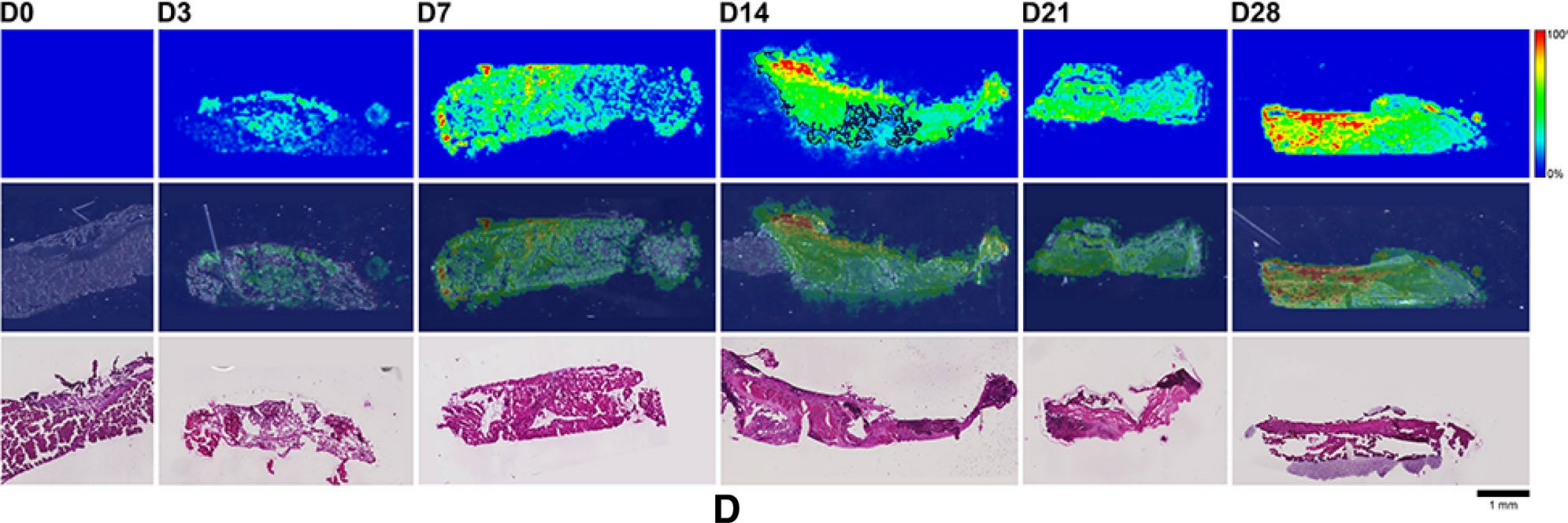

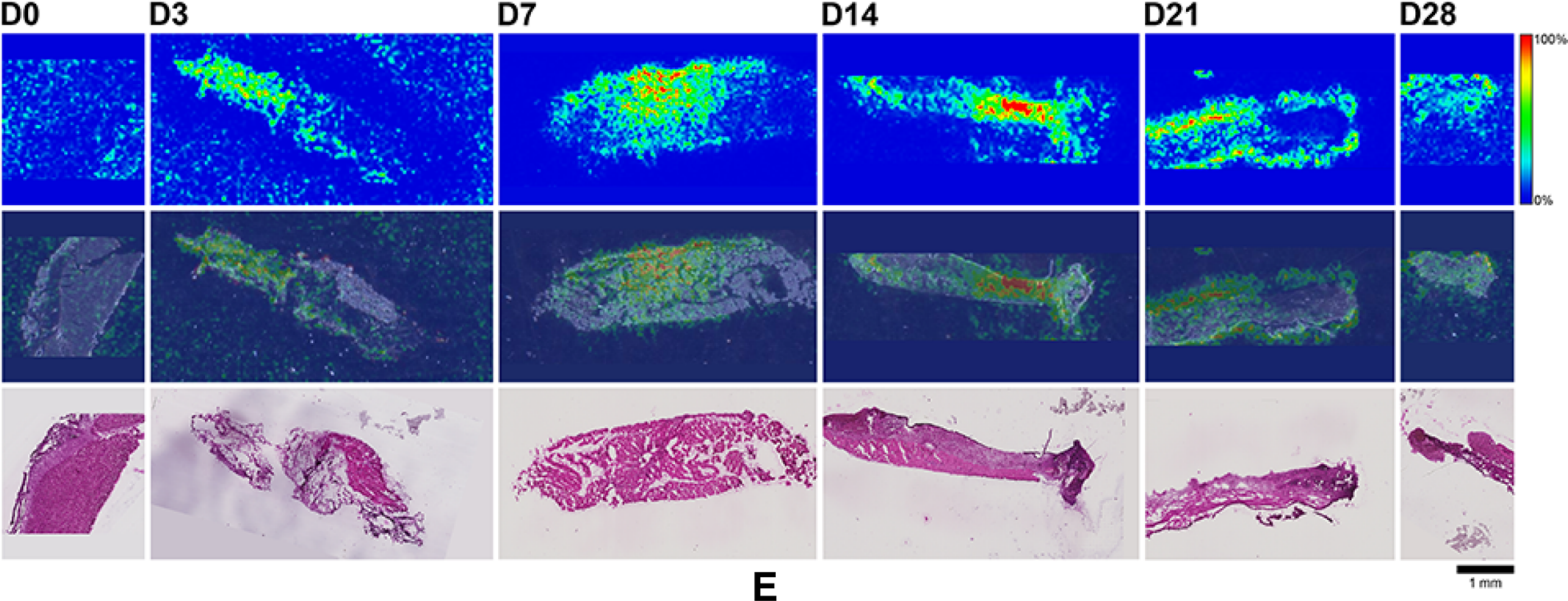

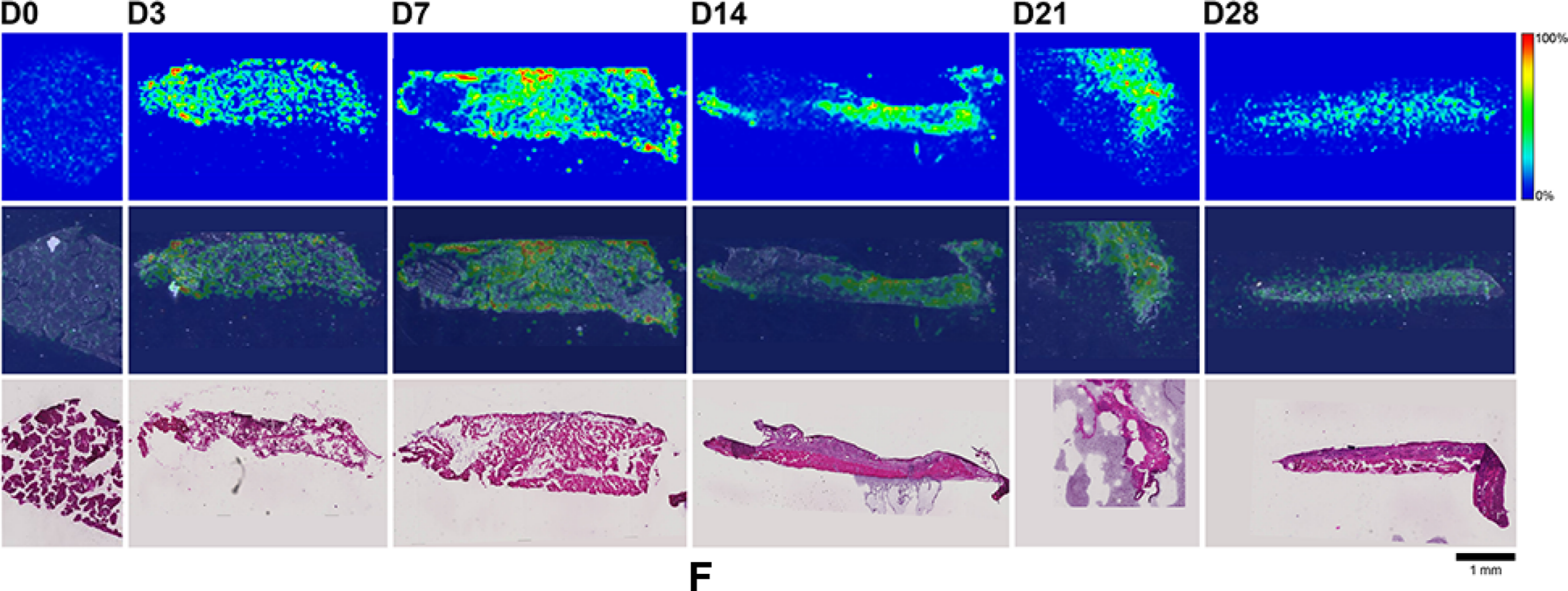
(**A**) Representative TAF spatial distribution profiles (positive mode, *m/z* 477 → 270, 346) in mouse dermal tissue sections adjacent to high-releasing (0.58 mg d^-1^) TAF implants; D0 indicates tissues from an unmedicated control; D3, D7, D14, D21, and D28 indicate tissue specimens collected with the TAF implant in place at study days 3, 7, 14, 21, and 28, respectively. The first row represents mass spectrometry images, generated at a spatial resolution of 50 µm and the color key in the legend indicates relative abundance, with red representing the highest signal intensity (100%) and blue representing the lowest signal (0%) for the measured ion; all images were scaled individually. The second row represents an overlay of the IMS signals and light microscopy images of the tissue specimens. The third row corresponds to H&E serial section stains of matching tissue specimens. Scale bar, 1 mm. (**B**). Representative Metabolite X spatial distribution profiles (positive mode, *m/z* 359 → 270, 288) in mouse dermal tissue sections adjacent to high-releasing (0.58 mg d^-1^) TAF implants. Additional details are the same as in (**A**). (**C**). Representative Metabolite Y spatial distribution profiles (positive mode, *m/z* 401 → 270) in mouse dermal tissue sections adjacent to high-releasing (0.58 mg d^-1^) TAF implants Additional details are the same as in (**A**). (**D**). Representative TFV spatial distribution profiles (negative mode, *m/z* 286 → 134, 151) in mouse dermal tissue sections adjacent to high-releasing (0.58 mg d^-1^) TAF implants. Additional details are the same as in (**A**). (**E**). Representative TFV-MP spatial distribution profiles (negative mode, *m/z* 366 → 268, 348) in mouse dermal tissue sections adjacent to high-releasing (0.58 mg d^-1^) TAF implants. Additional details are the same as in (**A**). (**F**). Representative TFV-DP spatial distribution profiles (negative mode, *m/z* 446 → 159, 348) in mouse dermal tissue sections adjacent to high-releasing (0.58 mg d^-1^) TAF implants. Additional details are the same as in (**A**).

**Figure 10:**
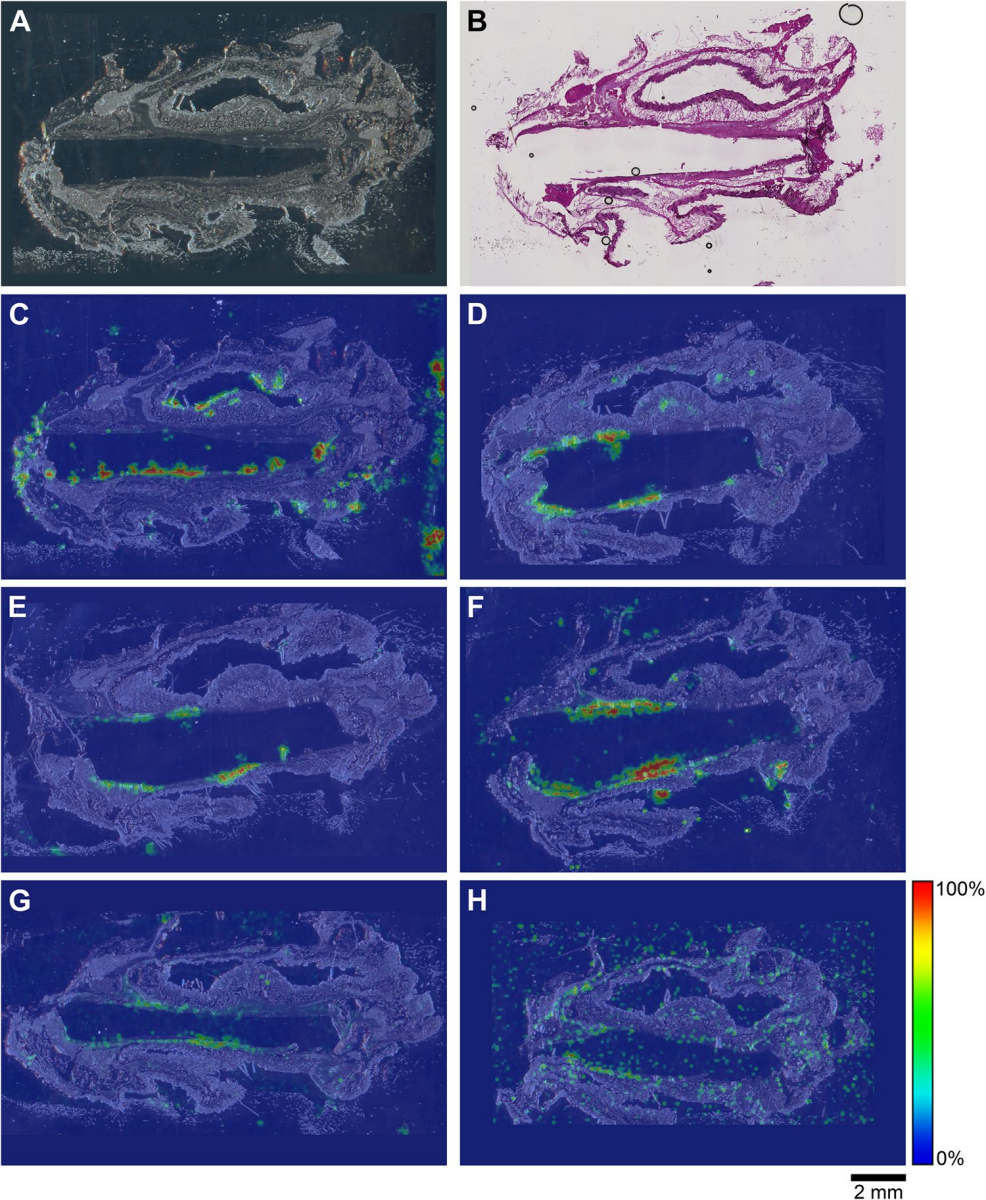
Representative TAF and drug metabolite spatial distribution profiles in mouse dermal tissue sections adjacent to low-releasing (0.23 mg d^-1^) TAF implants, with the implants remaining in situ within the encasing tissue block excised at necropsy, on study Day 14. (**A**) Light microscopy image of the tissue section. (**B**) H&E Serial section stain of matching tissue specimen. (**C**–**H**) Overlay of mass spectrometry images, generated at a spatial resolution of 100 µm, and light microscopy images of the tissue specimens. (**C**) TAF; (**D**) Metabolite X; (**E**) Metabolite Y; (**F**) TFV; (**G**) TFV-MP; and (**H**) TFV-DP. Color key in the legend indicates relative ion abundance, with red representing the highest signal intensity (100%) and blue representing the lowest signal (0%) for the corresponding measured ion; all images were scaled individually. Scale bar, 2 mm.

 Here, MALDI-IMS was used to visualize the distribution patterns of TAF and its main metabolites (Scheme 1) in dermal tissues adjacent to the implant. The MALDI-IMS analyses targeted TAF, Metabolite Y, Metabolite X, TFV, TFV-MP, and TFV-DP (Scheme 1) using sensitive and selective pseudo-selected reaction monitoring methods. A linear ion trap mass spectrometer equipped with a MALDI source and a nitrogen laser was used to detect the above compounds in dosed dermal (i.e., proximal to the implant) tissue sections. Optimal conditions are listed for the measured compounds in Supplementary Table 1. Representative mass spectrometry images showing the spatial distribution of TAF and its metabolites in dermal tissue sections over 28 days with the implant in place are shown in Figs. 9, 10, along with the corresponding hematoxylin and eosin (H&E) stained serial sections.

No (Fig. 9A–E) or very low (Fig. 9F) background signal due to the matrix and/or interfering species was observed in unmedicated (D0) samples. All six analytes were detected in all medicated specimens, but their spatial distribution in the tissue sections differed. The highly labile compounds TAF, Metabolite X, and Metabolite Y (Fig. 9A–C) generally were localized close to the implant, except on Day 28 when they appeared more diffuse. The three prodrug metabolites TFV, TFV-MP, and TFV-DP (Fig. 9D–F) were distributed homogeneously throughout the samples, but their concentration profiles were different. The images shown in Fig. 9 were acquired and intensity-scaled under different conditions. Consequently, the concentration of species cannot be compared directly using the color key. The data shown in Fig. 9 are from the high-releasing group, and tissue distribution profiles obtained for the low-releasing group are similar (Fig. 10).

The MALDI mass spectrometry images shown in Fig. 9 assume that the analyte concentration hot spots are adjacent to the TAF implant in vivo, located at the top of all the images, before it was excised at necropsy. Removal of the implants was necessary to measure their residual drug contents, and hence in vivo release rate, and care was taken to avoid disrupting the tissue specimen during removal. This was greatly facilitated by the gelatinous capsule surrounding the implant (vide infra) that could be cut longitudinally to allow the device to slide out easily using forceps.

In order to prevent potential bias resulting from implant removal, the study was repeated using low-releasing (0.23 mg d^-1^) implants described previously^32^ in C57BL/6 J mice. Here, the used implants were not removed from the tissue blocks, and the corresponding, representative MALDI-IMS data (Day 14) are summarized in Fig. 10.

The images shown in Fig. 10C–H with the implant in place are consistent with those shown in Fig. 9 (higher spatial resolution), where the implants had been removed from the tissue blocks for residual drug measurements prior to analysis. In Fig. 10, the implant is defined by the featureless rectangular area at the center of the image and the shape is a result of compression and sectioning angle. At lower resolution (Fig. 10, spatial resolution of 100 µm), the analytes consistently are concentrated around the perimeter defining the implant location. Unfortunately, concentration hot spots could not be related to the location of the implant delivery channels. However, the relative intensity and spatial distribution of the signals is heterogeneous and different across analytes. TAF and TFV resulted in the most abundant signals, although the latter appeared to have penetrated deeper into the tissues. Metabolites X and Y were localized close to the implant, with lower apparent abundances, as signal intensity needs to be interpreted with caution especially when comparing different analytes. Signals from TFV-MP and TFV-DP were weak, as expected from LC–MS/MS analysis of dermal tissue homogenate (TFV-DP). The apparent higher abundance of TFV-MP compared to the diphosphate could be related to ionization efficiency in the sample, not necessarily relative concentration.

### In vivo implant encapsulation

**Figure 11:**
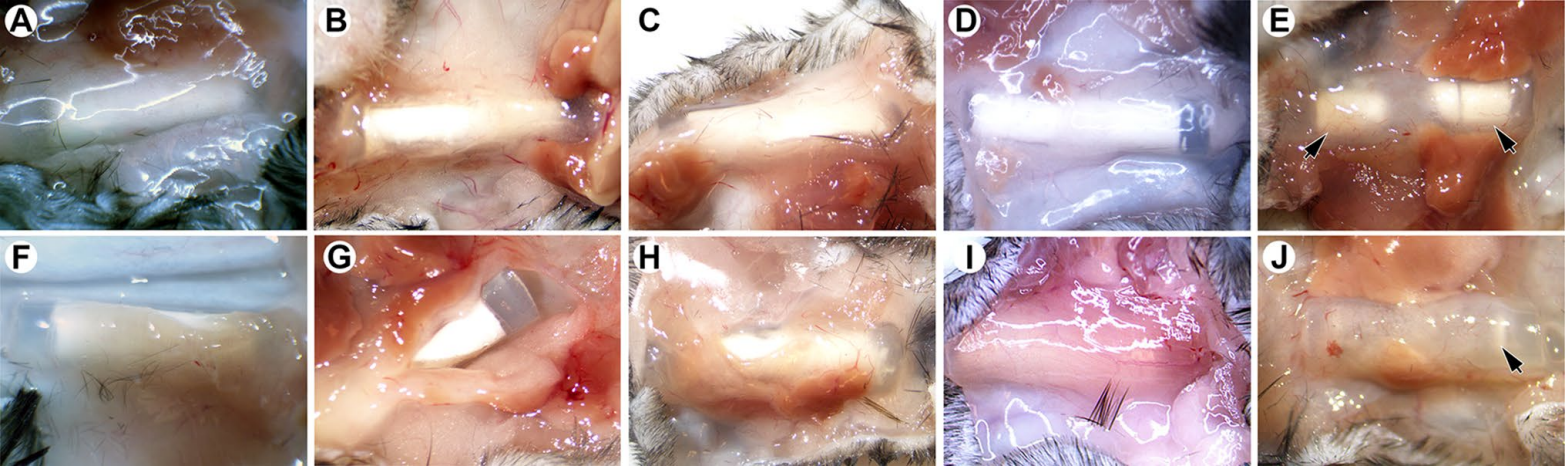
Images of used TAF implants at the time of excision from C57BL/6 J mice; (**A**–**E**), low-releasing; (**F**–**J**) high-releasing; (**A** and **F**), Day 3; (**B** and **G**), Day 7; (**C** and **H**), Day 14; (**D** and **I**), Day 21; and (**E** and **J**), Day 28; arrowheads identify TAF microtablets on study Day 28.

The implants were encapsulated in a gelatinous sheath at all timepoints in the mouse studies (Fig. 11).

Initial dissection of the implants from the mouse skin revealed little of importance related to adverse tissue reactions. The implants were present inside a thin transparent capsule, that remained unremarkable even after implants were in place for 28 days (Fig. 11E and J). Blood capillaries were observed invading the capsules around the implants as early as Day 3 after implantation (Fig. 11A and F). The capsules were sufficiently transparent to observe the TAF microtablets in the lumen of the implants, and the depletion of drug at Day 28, especially in the high-releasing group (Fig. 11J). Muscle tissue was attached to the outside surface of the capsule and was often removed during dissection.

### Local safety assessment

**Figure 12:**
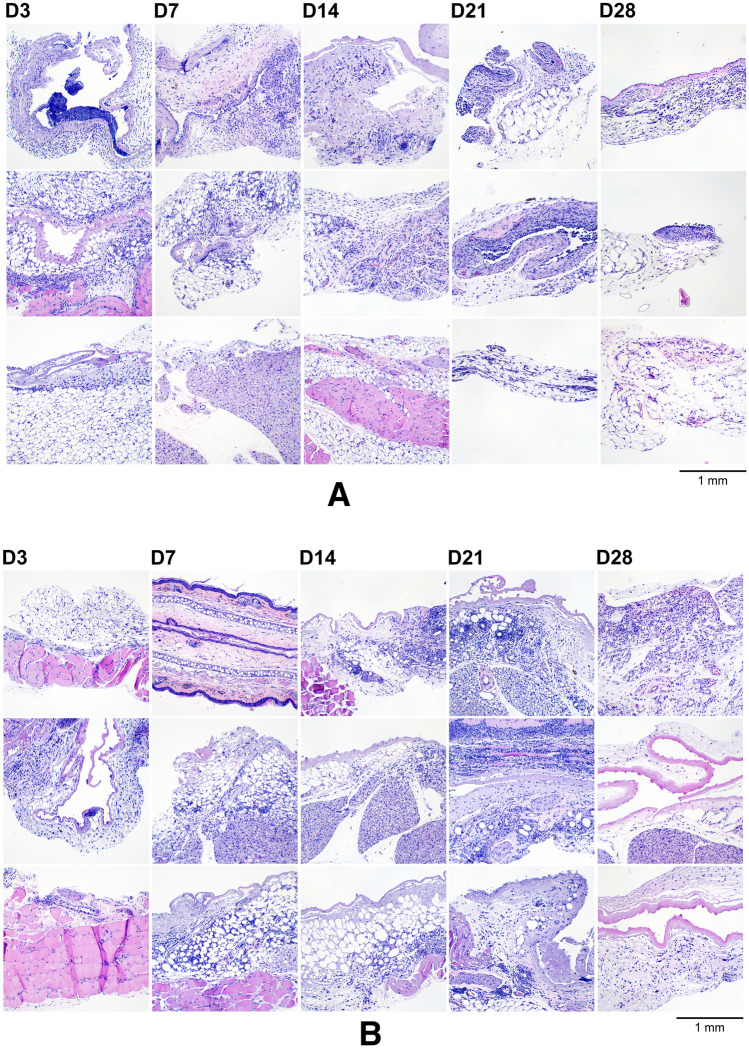
(**A**) Dermal tissues collected adjacent to the TAF implant from the low-releasing group, aldehyde-fixed, paraffin-embedded, sectioned, and H&E-stained. The specimens span the 28-day period where the implants were in place and representative samples from all animals (*N* = 3 per timepoint) are included; D3, D7, D14, D21, and D28 indicate tissue specimens collected with the TAF implant in place at study days 3, 7, 14, 21, and 28, respectively. Scale bar, 1 mm. (**B**) Dermal tissues collected adjacent to the TAF implant from the high-releasing group, aldehyde-fixed, paraffin-embedded, sectioned, and H&E-stained. The specimens span the 28-day period where the implants were in place and representative samples from all animals (*N* = 3 per timepoint) are included; D3, D7, D14, D21, and D28 indicate tissue specimens collected with the TAF implant in place at study days 3, 7, 14, 21, and 28, respectively. Scale bar, 1 mm.

**Figure 13:**
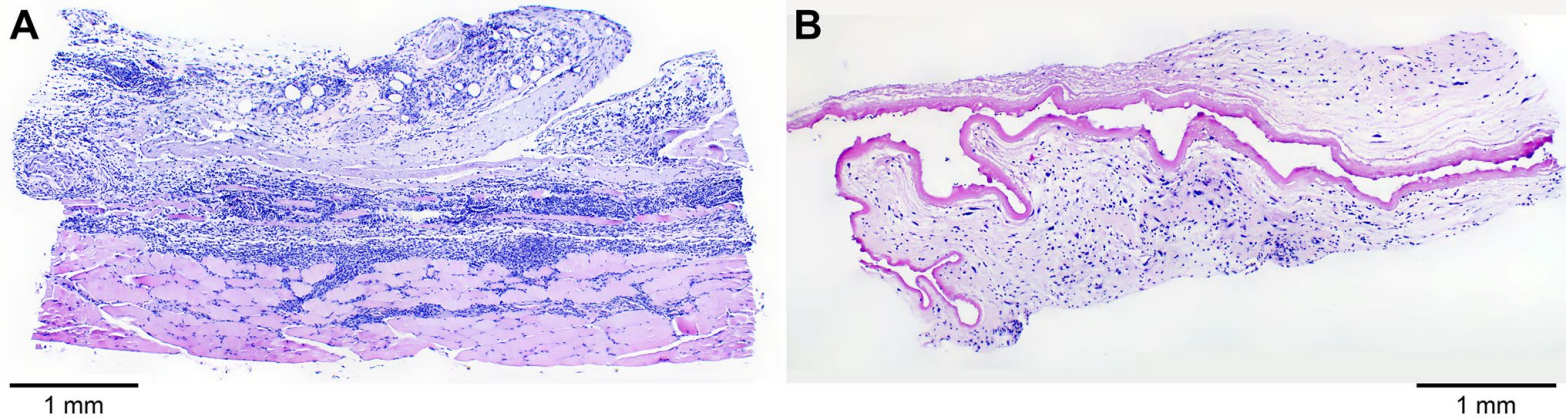
Light micrograph montages of H&E-stained mouse dermal tissue section images showing the capsules that surrounded the TAF implants at (**A**) 21 days and (**B**) 28 days. The tissue is folded with the capsule containing a lumen on one side and tissue on the other. (**A**) The capsule is surrounded by fat/fascia tissue (blue) and skeletal muscle (pink). (**B**) The capsule is surrounded by fat/fascia tissue. Scale bars, 1 mm.

No adverse events related to treatment with the test article were noted during the course of the mouse studies. Dermal tissue specimens collected adjacent to the implant at all timepoints were sectioned and H&E-stained for microscopic imaging (Figs. 12 and 13).

The H&E-stained sections shown in Fig. 12 contained variable amounts of skeletal muscle (stained pink), fat cells (blue), and a capsule that surrounding the implant prior to removal and consisted of a thin rim of eosinophilic, cell-poor fibrillar to hyaline material (purple/pink). The capsules sometimes were infiltrated by minimal amounts of granulation tissue at the periphery. In the fat/fascia, there was minimal mononuclear or mononuclear and neutrophilic inflammation without meaningful differences between groups (i.e., low- and high-releasing implants). Overall, there were no meaningful differences in the microscopic appearance of the tissues resulting from the low- and high-releasing TAF implant groups. Not all tissue types were present in all sections. Representative composite images, montages of individual H&E-stained tissue section images, covering the entire specimens (Fig. 13A, Day 21; Fig. 13B, Day 28) and showing the capsule that surrounded the implant prior to removal are presented below.

H&E-stained Day 28 dermal tissue specimens for all animals (*N* = 3 per group) in both implant groups (6 samples total) were submitted for clinical evaluation by a certified pathologist. The pathologist’s observations were in agreement with those presented above and no microscopic findings or meaningful differences between groups were noted. The pathologist concluded that there were no effects of TAF dose on the microscopic appearance of tissues from mice implanted with devices delivering low and high doses of TAF on Day 28. Histopathologic scoring of the tissue H&E-stained dermal tissue sections shown in Fig. 12 is presented in Table 3.

**Table 3:**
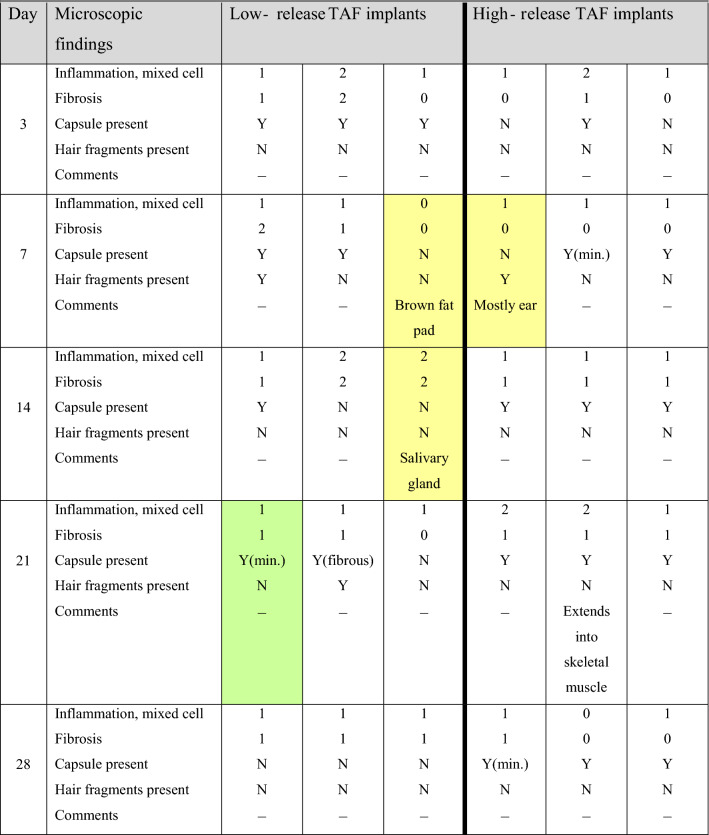
Histopathology-scored report of H&E-stained tissue sections of dermal tissues collected during subdermal TAF implant studies.

Definitions: *Inflammation,* mixed cell-inflammation with cellular infiltrates consisting of monocytes, macrophages (sometimes vacuolated), lymphocytes, and variable number of neutrophils. Cellular infiltrates were present in the white adipose tissue surrounding the capsule, within the outer capsule wall, and sometimes in the lumen of the capsule. *Fibrosis,* proliferation of fibroblasts (less mature, without much collagen) or increased mature fibroblasts and collagen; *Capsule,* thin strip of eosinophilic fibrillar material presumably surrounding implant; in one animal the capsule consisted of fibrous tissue (D21, “Low Release 1”, shaded green). Cells shaded yellow did not contain any capsule and consisted mostly of other tissues (e.g., brown fat, ear, salivary gland). The grading scale was between 1 and 5: 1, minimal; 2, mild; 3, moderate; 4, marked; 0, not present.

### Prophylactic efficacy against HIV-1 infection in bone marrow-liver-thymus (BLT) humanized (hu) mice following oral TAF dosing

**Figure 14:**
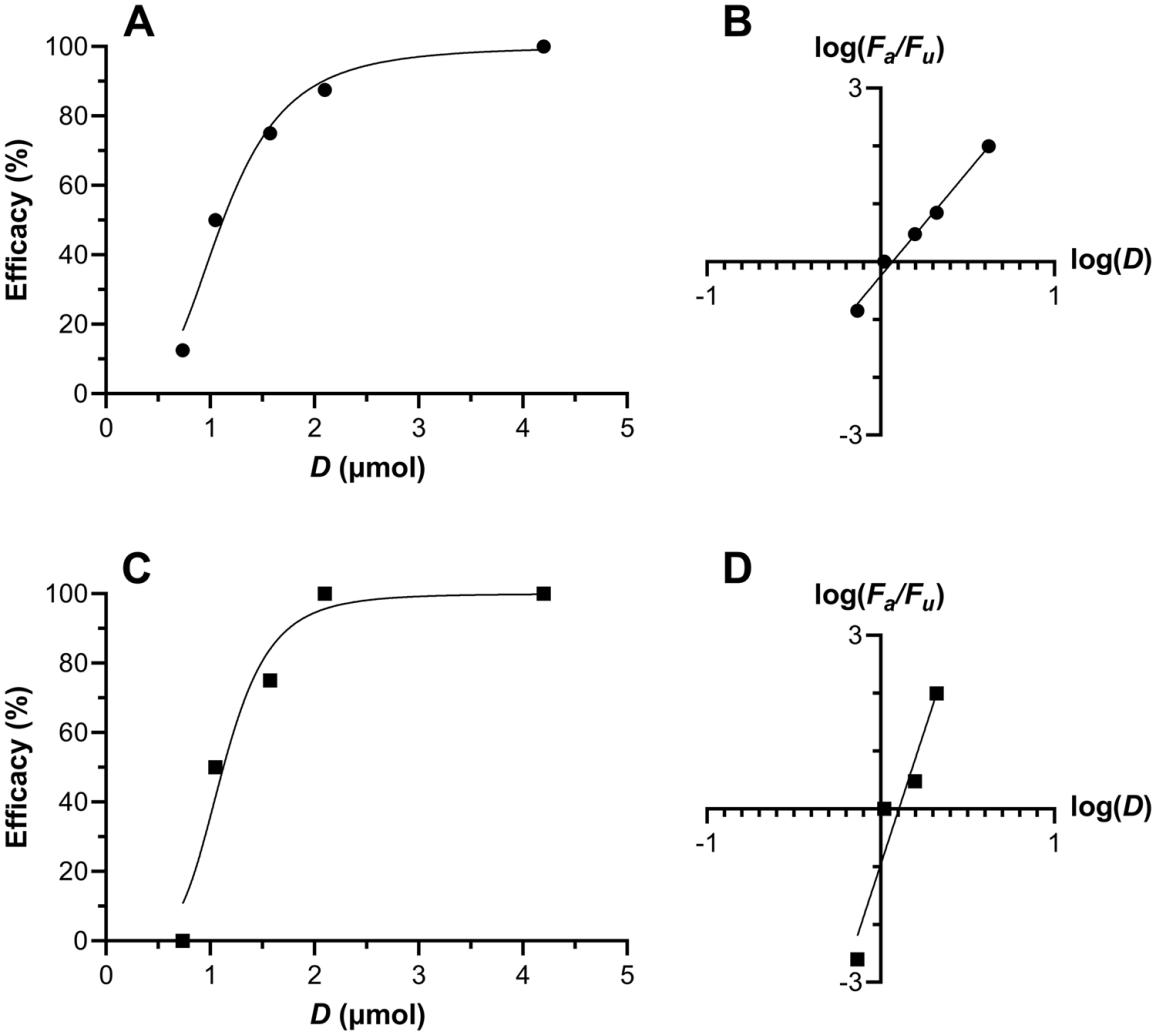
Dose–response curves for vaginal (**A**,**B**, circles) and rectal (**C**,**D**, squares) HIV-1 challenge studies in humanized BLT mice following oral TAF administration. Plots of (**A**) vaginal and (**C**) rectal efficacy *versus* dose of oral TAF (*N* = 8 per dosing group, 5 groups) administered 6 h prior to HIV-1 challenge. Solid lines are fits to a sigmoidal dose-normalized response (variable slope) model. Median-effect model analysis^59^ using log–log dose–response relationships of (**B**) vaginal and (**D**) rectal efficacy as a function of oral TAF dose allows key pharmacodynamic parameters to be calculated, as summarized in Table 4*F*_*a*_, fraction affected; *F*_*u*_, fraction unaffected; *D*, dose (µmol).

The BLT hu-mouse model has been used extensively in efficacy studies involving HIV-1 prevention research^54–58^. Here, oral TAF formulations of varying drug concentrations were administered to BLT hu-mice via gavage. The mice were challenged with HIV-1 either rectally or vaginally 6 h post dosing and monitored for infection status for 12 weeks. Results from these studies are presented in Fig. 14 and summarized in Table 4.

**Table 4:** Dose–response characteristics against HIV-1 infection of oral TAF in BLT hu-mice.

Pharmacodynamic parameter	Vaginal	Rectal
*ED*_50_ (µmol)	1.17	1.27
*ED*_90_ (µmol)	2.15	1.62
*m*-value ± SEM, *R*^2^	3.60 ± 0.22, 0.989	9.18 ± 1.94, 0.918

Using the calculated *ED* values in combination with our PK model (Fig. 7), target equilibrium plasma (TFV) and PBMC (TFV-DP) drug concentrations in C57BL/6 J mice associated with HIV-1 PrEP efficacy were calculated (Table 5). Oral bioavailabilities of TAF, *F*, were based on existing preclinical results^60^.

**Table 5:** Simulated systemic drug concentrations in C57BL/6 J mice at 6 h following oral TAF dosing over a range of oral bioavailabilities (*F*).

TAF dose µmol	*F*	Plasma [TFV] nM (ng mL^-1^)	PBMC [TFV-DP] nM (fmol/10^6^ cells)
1.25	0.1	0.451 (0.130)	38.0 (7.6)
1.25	0.25	1.13 (0.325)	94.5 (18.7)
2.00	0.1	0.723 (0.208)	60.5 (12.1)
2.00	0.25	1.82 (0.522)	147 (29.3)

## Discussion

Long-acting HIV-1 PrEP products will need to meet rigorous safety and efficacy endpoints in clinical trials before they become available for widespread use. Long-acting delivery of the potent prodrug TAF holds significant potential as a member of the HIV-1 PrEP product pipeline, prompting us^31,32,40^, and others^33–37,40,61,62^, to develop a range of innovative subdermal implant technologies. To date, our first-in-human clinical trial, CAPRISA 018^63^, conducted at multiple sites in South Africa is the only reported clinical evaluation of this approach. However, key questions surrounding the pharmacology of long-acting, subdermal TAF delivery and differing observations across research groups remain to be elucidated. The primary objectives of the current study were to address some of these knowledge gaps by investigating fundamental aspects of the PK (drug distribution over time) and pharmacodynamic (PD, drug biologic and physiologic effect; i.e., safety and efficacy) properties of subdermal TAF delivery via implant in mice. We have shown previously that, despite their small size, mice are a valid animal model for TAF implant preclinical evaluation^32^.

Efficacy studies in BLT hu-mice showed that oral TAF could be fully protective of vaginal and rectal HIV-1 acquisition. The drug exhibited similar potency in both compartments, although the dose–response relationship was steeper (higher *m*-value) for rectal HIV-1 prevention (Table 4). Using our PK model (Fig. 7) we predicted the PBMC TFV-DP concentrations in C57BL/6 J mice, the species used for the preclinical implant safety and PK studies, associated with 90% protection from HIV-1 infection in the 60.5–147 nM (12.1–29.3 fmol/10^6^ cells) range (Table 5), suggesting that both implant types would be protective of HIV-1 infection in mice based on the measured TFV-DP PBMC concentrations (Fig. 5B). These values are remarkably consistent with HIV-1 PrEP clinical efficacy data from iPrEX, a randomized clinical trial involving HIV-negative MSM on a daily oral regimen consisting of TDF and FTC. HIV-1 protection efficacy was found to be 92% in moderately adherent participants, as evidenced by plasma TFV concentrations^14^. Importantly to the current report, a post hoc analysis of iPrEX data found that a PBMC TFV-DP concentration of 16 fmol/10^6^ cells was associated with 90% protection ^64^. However, the trial used cryopreserved PBMCs, subsequently shown to result in a 33 to 67% TFV-DP loss^31^. Because the PBMCs were lysed here prior to freezing, there-by preserving the TFV-DP, a more conservative TFV-DP EC_90_ lies in the 24 to 48 fmol/10^6^ cells range^31^. Our modeled PBMC TFV-DP data associated with 90% protection from vaginal and rectal HIV-1 prevention in mice falls within this range. To our knowledge, this is the first example of PK-PD relationships in the mouse model agreeing with clinical HIV-1 PrEP data.

The relationship between SQ TAF administration and the concomitant plasma TFV concentration–time profile is complex (Scheme 1). Non-linear processes are possible at the dosing site, as TAF is cleared from the subcutaneous pocket either by migration (via passive diffusion and possibly active transport) to other anatomic locations or TAF metabolizes to intermediate species (see Figs. 9 and 10). Because these processes are rapid and the intermediates unstable (i.e., challenging to quantify by LC–MS/MS), the nature of these non-linear processes can be difficult to characterize. Pharmacokinetic modeling in C57BL/6 J mice resulted in the surprising finding that TAF exposure scaling (i.e., apparent bioavailability) from subdermal implants was 4–5 × higher, in a dose-dependent manner (higher exposure scaling at higher implant TAF release rates), than when TAF was administered by SQ or IV injection. The increase in exposure scaling during low, continuous TAF delivery compared to high, bolus dosing appears to be a real physiologic phenomenon. While rare, bioavailabilities exceeding 100% in the presence of complex absorption processes have been reported for a vitronectin receptor antagonist (SB-265123)^65–67^. In their mechanistic PK modeling rat studies, Ward et al. concluded that saturable active transport processes were responsible for an apparent oral *F* > 100%^67^.

Saturable processes, either mediated by molecular transporters or enzymatic metabolism, are a possible explanation for the high exposure scaling observed here for the TAF implants. While TAF is not believed to be a substrate for renal uptake transporters (OAT1 and OAT3) based on in vitro studies^68^, the prodrug appears to be vulnerable to efflux transport in the intestine^60^. Saturable, transporter-driven processes between TAF diffusion from the implant to the subcutaneous milieu through the cascade of processes culminating with intracellular metabolism (Scheme 1) cannot be ruled out as a possible explanation for the observed phenomenon. Birkus et al. have shown that intracellular TAF activation is mediated by lysosomal serine protease cathepsin A (CatA)^51,52^, and other human hydrolases also are implicated in TAF metabolism^53^. Following hydrolysis of the *iso*-propyl ester (step 1 in Scheme 1), likely by a cellular hydrolase including CatA, the phosphonoamidate prodrug spontaneously eliminates the phenol moiety (step 2 in Scheme 1) to afford Metabolite X via a cyclic intermediate (step 3 in Scheme 1). Deamination of Metabolite X yields TFV (step 5 in Scheme 1)^51^, and sequential phosphorylation by intracellular AMP kinase and nucleoside diphosphate kinase form TFV-DP (steps 6 and 7 in Scheme 1)^69^, the active metabolite against HIV-1 reverse transcriptase. The many saturable enzymatic steps involved in the formation of intracellular TFV-DP from TAF also could form part of the explanation for high observed implant exposure scaling.

An alternative explanation is that flip-flop kinetics in the implant groups (unlike the IV and SQ injection studies where flip-flop kinetics were not observed) in the absorption of a highly water-soluble compound with rapid systemic clearance are responsible for the unexpected exposure scaling. In other words, a slow parenteral release may “shield” TAF and its metabolites from the rapid clearance of the high-capacity liver. Irrespective of the mechanism(s), it is clear that if this phenomenon translates to humans at the target implant TAF release rate, it may lead to longer than previously anticipated in vivo duration of use, compared to estimates based on bolus dosing.

Drug and drug metabolite distribution in tissues of pharmacologic importance for HIV-1 PrEP was investigated thoroughly in C57BL/6 J mice following parenteral TAF dosing. High-releasing TAF implants resulted in consistently higher plasma TFV, PBMC TFV-DP, and dermal tissue (adjacent to the implant) TFV and TFV-DP concentrations than low-releasing TAF implants (Fig. 5A–D). The mole fraction of TFV-DP, *x*(TFV-DP), in tissues surrounding the implants for the high- and low-releasing implant groups was not found to be significantly different (*P* = 0.9349) using a Mann–Whitney *t*-test (Fig. 6B). Dermal tissues also contained the lowest TFV-DP mole fraction for all tissue types examined, with median *x*(TFV-DP) values of 0.0044 and 0.0065 for low- and high releasing implant groups, respectively, indicating that less than 1% of the total TFV species on a molar basis was present as TFV-DP. In contrast, tissue homogenate samples from rectal, spleen, and thymus –compartments rich in immune cells– collected following IV TAF administration exhibited *x*(TFV-DP) values exceeding 0.2 (TFV-DP made up > 20% of the total TFV species on a molar basis), while the median value in vaginal tissue homogenate was 4.7–6.7-fold lower, depending on the compartment (Fig. 6A). These results together with the consistently higher TFV-DP PBMC concentrations compared with plasma TFV (e.g., Fig. 5A–B) indicate that TAF is fulfilling its promise by efficiently loading immune cells with active metabolite, TFV-DP.

The spatial distribution of TAF and its metabolites in dermal tissues surrounding the implant is important when interpreting local tolerance findings. Traditional bioanalytical methods (e.g., LC–MS/MS) provide accurate, aggregate measurements of the analyte concentration in a sample, but do not provide information how the compound is distributed spatially within the specimen. Additionally, analyte concentration hot-spots within a sample can be lost (i.e., below the lower limit of quantification) in an assay that integrates the measurement over the entire sample, depending on the sensitivity of the assay. Orthogonal technologies such as LC–MS/MS and MALDI-IMS are helpful in characterizing the samples in terms of accurate, sensitive, integrated drug concentrations for PK-PD analyses (LC–MS/MS) and as spatial analyte distribution to understand more nuanced drug localization (MALDI-IMS). To complement traditional LC–MS/MS approaches, imaging mass spectrometry has been applied to visualize ARV drug distribution in a variety of sample types following bolus dosing^70–74^. These studies elegantly showed the usefulness of MALDI-IMS in the context of HIV/AIDS prevention and treatment research. Ours is the first account where this powerful technique has been applied longitudinally to investigate drug and drug metabolite distribution in a long-acting drug delivery context (Fig. 9). Dermal tissue homogenate analysis by LC–MS/MS provided integrated drug concentrations across the entire specimen, but the spatial information on the analytes was lost and unstable compounds (e.g., TAF, Metabolite X, and Metabolite Y) were metabolized during sample preparation. All analytes of interest (i.e., TAF, Metabolite X, Metabolite Y, TFV, TFV-MP, and TFV-DP) were detected by MALDI-IMS in all dermal tissue samples analyzed during implant TAF delivery (Figs. 9 and 10). These results constitute the first report of Metabolite X and Y in vivo detection following TAF dosing. While all analytes were located primarily adjacent to the perimeter defined by the implant, their distribution was heterogeneous as was the extent of tissue penetration.

Implants rapidly became embedded in a clear, vascularized, gelatinous capsule (Fig. 11) following placement in vivo. The low- and high-releasing implants were well tolerated over the 28-day period of use, and local effects were characterized by the expected foreign body response^75^ and no concerning adverse safety findings (Figs. 12, 13). Selecting the C57BL/6 J mouse model for PK and local safety studies largely was motivated by the knowledge that this species has a foreign body response to implanted devices similar to human ^76^. Our local tolerance observations are in general agreement with those found by others delivering TAF hemifumarate from a nanofluidic implant in rhesus macaques^34,62^ for up to 70 days^34^. Longer studies will be required in the future to further evaluate the safety of our TAF implants.

In a separate study, Su et al. reported concerning local toxicity in NZW rabbits and rhesus macaques when delivering TAF hemifumarate from a polyurethane implant^36^. The safety findings ranged from local inflammation to severe tissue necrosis and were greater in the medicated group suggesting a drug-related effect, although two of four macaques with both placebo and medicated implants placed contralaterally for 90 days also showed extensive inflammation in the placebo groups. The toxicity was greater in macaques than in rabbits and was evident after 4 weeks of implant use, but became even more pronounced at 12 weeks.

The local tissue TFV-DP concentrations at the implant site in rabbits were highly variable in the report by Su et al.^36^. For low-releasing implants (0.13 and 0.26 mg d^-1^ in vitro) at 4 weeks, the TFV-DP tissue homogenate concentrations generally were below 5 fmol mg^-1^, but at 12 weeks they were distributed over three orders of magnitude between the extremes of 86 and 70 × 10^3^ fmol mg^-1^. High-releasing implants (0.48 and 0.72 mg d^-1^ in vitro) already exhibited high variability in dermal tissue TFV-DP concentrations after 4 weeks (range 32–12 × 10^3^ fmol mg^-1^) as well as 12 weeks of use (range 28–45 × 10^3^ fmol mg^-1^). No corresponding tissue TFV concentrations were reported. Surprisingly, when implants releasing 0.13 mg d^-1^ TAF in vitro were evaluated in macaques, “all TFV concentrations near the implant site were BLQ (LLOQ = 0.05 ng/sample)”^36^, while the corresponding TFV-DP concentrations also were BLQ or low (1.1–27 fmol mg^-1^) after 12 and 13 weeks of use. Because the toxicity in macaques was worse than rabbits, but far lower local TFV and TFV-DP concentrations were measured, the toxicity cannot be attributed to either TFV or TFV-DP. In our TAF implant studies, dermal tissue TFV-DP made up less than 1% of the total TFV species on a molar basis and median concentrations for the low- and high-releasing implants were 1.4 × 10^3^ and 6.3 × 10^3^ fmol mg^-1^, respectively (Table 1), suggesting that different metabolic processes could be occurring adjacent to the implants.

It is possible that mechanical trauma from implantation and the physical characteristics of the device, together with the chemical composition of the polyurethane shell, generated an inflamed local environment –as observed in some of the placebo groups– that was further aggravated by TAF or one of its metabolites in combination with fumaric acid, since the hemifumarate salt is used not the free-base as in our studies. These differing observations across technologies highlight the complexity of long-acting parenteral TAF delivery necessitating an understanding of the mechanisms that underpin the associated PK and PD processes.

The studies described here have met their objectives in elucidating the pharmacology underlying sustained TAF delivery from subdermal implants in mice. The experimental approaches and results are transferrable to others working in the field. We conclude that because of its potency and targeted in vivo distribution, TAF remains a viable drug for parenteral long-acting delivery and merits further preclinical and clinical evaluation.

## Methods

### Materials

TAF, as the free-base, was kindly provided by Gilead Sciences, Inc. (Foster City, CA). Medical-grade silicone tubing was custom-manufactured by Trelleborg Healthcare & Medical (Los Robles, CA). All other chemicals and reagents were purchased as described previously^31^, unless otherwise noted.

### TAF Implant fabrication

Mouse-sized (length, 10 mm) TAF implants were fabricated using methods described previously^31^. In the current study, TAF was compacted into microtablets without the addition of excipients using a pellet press (Globe Pharma MTCM-I, North Brunswick, NJ)^77,78^. Each implant contained a median dose of 24.7 mg TAF. Implants were fabricated in a low bioburden environment and were cleaned with 70% v/v isopropanol using a sterile cotton swab, prior to being sealed individually in moisture-barrier pouches.

### Animal care and ethics statement

All mouse studies were carried out at The Scripps Research Institute (Permit Number: 13-0001) in strict accordance with the recommendations in *the Guide for the Care and Use of Laboratory Animals of the National Institutes of Health*^79^, under approved internal Institutional Animal Care and Use Committee protocols using internal Standard Operating Procedures. Pharmacokinetic studies were carried out with C57BL/6 J mice, while efficacy studies were carried out with bone marrow-liver-thymus (BLT) humanized (hu) mice. These mice were maintained and efficacy studies were performed in animal biosafety level 3 facilities at the Department of Animal Resources (DAR), at The Scripps Research Institute. All surgery was performed under anesthesia using 0.2 mL of 10% ketamine and 4% xylazine in phosphate-buffered saline (PBS), and all efforts were made to minimize suffering. Cervical dislocation was used as the method of sacrifice*.*

### Intravenous TAF pharmacokinetic mouse study design

The study product consisted of a TAF solution (5 mg mL^-1^) in the following vehicle: ethanol (5% v/v), polyethylene glycol (PEG) 300 (30% v/v), and water (65% v/v). The TAF solution was prepared less than 2 h before dosing. C57BL/6 J Mice (9 groups of 4 animals each; 36 mice total) were administered the study product (0.5 mg dose, 0.1 mL) by intravenous (IV) injection. Animals (*N* = 4) were sacrificed at the following timepoints: Day 1 (1, 3, 6, 12, 24 h), Day 2, 4, 8, and 14. Blood was collected by exsanguination along with select tissue specimens (vide infra).

The isolation of plasma and PBMCs from blood samples was carried out in a laminar flow hood using aseptic techniques, and the entire process was completed in under 45 min to minimize analyte metabolism. Blood was transferred into collection tubes containing sodium citrate (final concentration, 0.4% w/v), diluted using one volume equivalent of 1 × PBS solution, and mixed by inversion. The minimum final volume was 4.0 mL. Prior to the addition of blood samples, Ficoll-Paque media (MilliporeSigma, Burlington, MA, 3.2 mL) was transferred into a 12 mL LeucoSep tube (Greiner, Monroe, NC) and centrifuged at 1000 g for 20 s at room temperature, causing the media to settle just below the porous barrier. The homogenous, diluted blood-PBS mixture was transferred into the prepared LeucoSep tube and centrifuged at 1000 g for 10 min at room temperature, resulting in the formation of three layers above the porous barrier: (A) top layer, consisting of the plasma fraction; (B) middle, interface layer, containing the PBMCs; and (C) thin lower layer, just above the barrier, consisting of Ficoll-Paque media. Layer A (plasma) was transferred to empty 1.5 mL microfuge tubes in 0.5 mL aliquots, and flash-frozen in liquid nitrogen. Layer B (PBMCs) was transferred into an empty 15 mL centrifuge tube and PBS added to the 12 mL mark, followed by gentle inversion mixing. The suspension was centrifuged at 400 g for 15 min, the supernatant removed, and the pellet resuspended in PBS (10 mL) by gentle pipetting. An aliquot (10 µL) was removed from the suspension for cell counting and the remaining sample was centrifuged at 400 g for 15 min. The supernatant was removed without disturbing the cell pellet, which was treated with ice-cold 70% v/v methanol in molecular-grade water (1.0 mL). The mixture was mixed lightly by vortex agitation to lyse the cells, followed by transfer to a 2.0 mL cryovial that was flash-frozen in liquid nitrogen.

Vaginal, rectal, spleen, and thymus tissue samples were collected from sacrificed animals at Day 1 (12, 24 h), Day 4, and 8; rectal tissue samples were not collected at 12 h. The samples were transferred into pre-weighed sample tubes, weighed, and flash-frozen in liquid nitrogen.

All samples were stored and transported at − 80 °C prior to analysis.

### Subcutaneous TAF dosing pharmacokinetic mouse study design

C57BL/6 J Mice (12 groups of 4 animals each; 48 mice total) were administered the study product (0.5 mg dose, 0.1 mL) described above by subcutaneous (SQ) injection. Animals (*N* = 4) were sacrificed at the following timepoints: Day 1 (1, 3, 6, 12, 24 h), Day 2, 4, 6, 8, 10, 12, and 14. Blood was collected by exsanguination and processed as described above.

### Subcutaneous TAF implant study pharmacokinetic mouse study design

TAF implants were surgically implanted at the backside of the vertebral column in the dorsal scapular region of C57BL/6 J mice^32^. Two study groups are described here: one low-releasing and one high-releasing implant group. Animals (*N* = 3 per implant group) were sacrificed at the following timepoints: Day 3, 7, 14, 21, and 28 (15 mice total per implant group). Blood was collected by exsanguination and plasma and PBMC samples were processed as described above.

The used implants, the surrounding capsule, and the encasing dermal tissue were removed as a block in accordance with ISO 10993-6 guidelines^80^, processed and stored, as described elsewhere^32^. Briefly, the capsule was cut longitudinally and rolled open, taking care not to disrupt the architecture of the tissue or to damage the used implant. A portion of the tissue specimen was placed into 4% paraformaldehyde solution (phosphate-buffered at pH 7.2), and stored at 4 °C, for histopathology and two other portions were flash-frozen in liquid nitrogen for drug concentration analysis as well as for drug spatial distribution analysis by MALDI-IMS (vide infra).

In a separate study, low-releasing TAF implants from a different fabrication lot were evaluated in C57BL/6 J mice as described above except that spleen and thymus tissues also were collected at sacrifice on Day 3, 7, 14, and 28. These specimens were flash-frozen in liquid nitrogen for drug concentration analysis.

In another study, TAF implants from a fabrication lot described previously^32^ were evaluated in C57BL/6 J mice as described above except that the used implants were left encased in their dermal tissue blocks, which were flash-frozen in liquid nitrogen at sacrifice for analysis by MALDI-IMS.

### In vivo TAF release rates

Residual drug analysis in used implants was used to determine in vivo TAF release rate according to published methods^31^.

### Bioanalysis methods

Drug concentrations in plasma (TAF, TFV) and tissue homogenate (TFV, TFV-DP) samples were measured using liquid chromatography-tandem mass spectrometry (LC–MS/MS) according to methods described in detail elsewhere^31,81^. Mouse plasma from the implant groups was analyzed at Oak Crest with lower limits of quantification (LLQ) for TAF and TFV in plasma of 0.5 and 5 ng mL^-1^, respectively. The remaining samples were analyzed by the Clinical Pharmacology Analytical Laboratory at the Johns Hopkins University School of Medicine with the following LLQs: plasma: TAF, 0.03 ng mL^-1^; TFV, 1 ng mL^-1^; PBMCs: TFV-DP, 5 fmol/sample; dermal tissue: TFV, 0.05 ng/sample; TFV-DP, 5 fmol/sample. PBMC results were normalized to the number of cells and reported as fmol/10^6^ cells or as intracellular concentration based on cell volume, in nM. Tissue results were normalized to sample mass and reported as ng mg^-1^ or fmol mg^-1^.

### Pharmacokinetic data analyses

Compartmental analyses were performed in Phoenix software (version 8.3, Certara, Princeton, NJ) using a published, simple, structural PK model describing the TFV kinetics^44^. IV and SQ data were modeled simultaneously. Due to the destructive nature of the blood sampling, data were modeled using a naïve-pooled algorithm, without inter-animal variability. The resulting systemic parameters, along with the implant in vivo release rates on a per animal basis, were used to co-model (predict) concentration data in mice following during implant dosing.

### Tissue sample preparation for imaging mass spectrometry analysis

Frozen mouse tissue specimens were shipped to Vanderbilt University on dry ice and stored at − 80 °C prior to use. Tissues were removed from the − 80 °C freezer and placed in a − 20 °C cryostat (CM 1900, Leica Biosystems, Buffalo Grove, IL) and sectioned. Tissue samples that had been prepared via removal of the used implant were affixed to the cryostat chuck with optimal cutting temperature (OCT) polymer and were cut on the cryostat into 12 µm thick sections for imaging mass spectrometry and H&E staining.

Tissue samples that still contained the implant in place were embedded in OCT prior to sectioning. In order to minimize the artifactual signal from the TAF powder after sectioning (the act of the cryostat blade cutting through an intact implant distributed residual TAF powder that still remained in the used implant onto the surrounding tissue section), house vacuum was used to remove visible TAF powder, followed by lightly running nitrogen gas over the plate to remove any additional loose powder from the slide.

The tissue sections were thaw-mounted onto indium-tin oxide coated glass slides for imaging (Delta Technologies, Ltd, Loveland, CO). The tissue sections were left to dry for at least 15 min in a desktop vacuum desiccator prior to matrix application. 9-Aminoacridine (9AA, 5 mg mL^-1^ in 90% v/v methanol) was used as the matrix for the analysis of TFV, TFV-MP and TFV-DP, while 2,5-dihydroxybenzoic acid (DHB, 40 mg mL^-1^ in 70% v/v methanol with 0.1% v/v trifluoroacetic acid) was used for the analysis of TAF, Metabolite X, and Metabolite Y. Both matrices were applied to the tissue sections using an automated sprayer (TM Sprayer, HTX Technologies, Chapel Hill, NC). For 9AA, four passes were applied at 85 °C in a crisscross pattern, with a 700 mm min^-1^ stage velocity, 2 mm track spacing, and 0.15 mL min^-1^ flow rate. For DHB the same method was used, except eight passes were applied and the stage velocity was 1300 mm min^-1^.

### Matrix-assisted laser desorption/ionization (MALDI) imaging mass spectrometry (IMS) analysis of dermal tissue specimens

Flash-frozen dermal tissue samples collected adjacent to the TAF implants and sectioned were analyzed with a linear ion trap mass spectrometer equipped with a MALDI source and a nitrogen laser (LTQ XL, Thermo Scientific, Waltham, MA). The structures of compounds of interest are shown in Scheme 1. Targeted MS/MS methods were optimized for each compound using authentic samples as standards, with the final parameters shown in Appendix A under Supplementary Information. Pseudo-selected reaction monitoring mode was used for imaging by isolating precursor ions with a 1 Da window, fragmenting them, and acquiring full product ion mass spectra at each pixel. TFV, TFV-MP, and TFV-DP were analyzed in negative ion mode, while TAF, Metabolite X, and Metabolite Y were analyzed in positive ion mode. In most cases, after imaging, the matrix was removed and the tissue section that had been imaged was H&E stained for better image registration with tissue morphology. Standard images were acquired at either 50 or 100 µm spatial resolution. Reconstructed ion images were generated in ImageQuest software (Thermo Scientific, Waltham, MA) by plotting the intensity of the diagnostic fragment ion or ions as a function of location across the tissue surface.

### Assessment of local safety

Toxicity was evaluated by clinical observations, cage-side observations (twice daily), and body weight (weekly). Formaldehyde-fixed dermal tissue specimens (one per animal) were paraffin-embedded, sectioned, and H&E stained using established methods (JIT Labs, jit-labs.com). The slides were evaluated for microscopic findings by a certified pathologist (Vet Path Services, Inc., Mason, OH). Histopathology grades were assigned as grade 1 (minimal), grade 2 (mild), grade 3 (moderate), grade 4 (marked), or grade 5 (severe) based on an increasing extent of overall change.

### Prophylactic efficacy against HIV-1 infection in bone marrow-liver-thymus (BLT) humanized (hu) mice following oral TAF dosing

The generation of BLT hu-mice was performed as described in Baum et al.^58^, and the references contained therein.

Efficacy studies were performed by administering BLT hu-mice (*N* = 8 per dosing group; 5 dosing groups per arm; two arms; 80 mice total) a freshly prepared TAF (0.35, 0.50, 0.75, 1.00, or 2.00 mg corresponding to 0.73, 1.05, 1.57, 2.10, and 4.20 µmol) solution in 50 mM citric acid (pH 5.0) via oral gavage. Vaginal or rectal challenge with HIV-1 was performed 6 h after dosing and HIV-1 infection status was monitored by quantifying viral RNA concentration in peripheral blood (plasma) at 1, 2, 3, 6, and 12 weeks post challenge as fully described in Baum et al.^58^, and the references contained therein.

### Data visualization and analysis

Data were analyzed and plotted in GraphPad Prism (version 9.2.0, GraphPad Software, Inc., La Jolla, CA). Statistical significance is defined as two-sided *P* < 0.05. The unpaired, nonparametric (i.e., do not assume a Gaussian distribution) Mann–Whitney test was used to compare two groups. The nonparametric Kruskal–Wallis tests with no matching/pairing of the data was used to compare three, or more groups. Images were compiled into figures using Adobe Photoshop CS6 (version 13.0, Adobe Systems, Inc., San Jose, CA). Image modifications consisted of brightness and contrast adjustments applied to the whole image. Image cropping was performed for presentation purposes.

## Supplementary Information

Supplementary Information.

## Data Availability

All other data supporting the findings of this manuscript are available from the corresponding author (MMB) upon reasonable request.

## References

[1] UNAIDS. 90-90-0: An Ambitious Treatment Target to Help End the AIDS Epidemic. Report No. UNAIDS/JC2684, 40 (UNAIDS, Geneva, CH, 2014).

[2] UNAIDS. Prevailing against Pandemics by Putting People at the Centre—World AIDS Day Report 2020. Report No. UNAIDS/JC3007E, 92 (UNAIDS, Geneva, CH, 2020).

[3] UNAIDS. *Global Factsheet 2017*, <AIDSinfo.unaids.org> (2018).

[4] Javanbakht M (2010). Prevalence and correlates of heterosexual anal intercourse among clients attending public sexually transmitted disease clinics in Los Angeles County. Sex. Transm. Dis..

[5] Gorbach PM (2014). Order of orifices: Sequence of condom use and ejaculation by orifice during anal intercourse among women: implications for HIV transmission. J. Acquir. Immune Defic. Syndr..

[6] Owen BN (2015). Prevalence and frequency of heterosexual anal intercourse among young people: A systematic review and meta-analysis. AIDS Behav..

[7] Hendrix CW (2018). HIV antiretroviral pre-exposure prophylaxis: Development challenges and pipeline promise. Clin. Pharmacol. Ther..

[8] Nuttall J (2012). The Pharmacokinetics of tenofovir following intravaginal and intrarectal administration of tenofovir gel to rhesus macaques. Antimicrob. Agents Chemother..

[9] Hendrix CW (2013). MTN-001: Randomized pharmacokinetic cross-over study comparing tenofovir vaginal gel and oral tablets in vaginal tissue and other compartments. PLoS ONE.

[10] Holt JD (2015). The sheep as a model of preclinical safety and pharmacokinetic evaluations of candidate microbicides. Antimicrob. Agents Chemother..

[11] Nair, G. *et al.* in *8th IAS Conference on Pathogenesis Treatment & Prevention.* Abstract # TUACO206LB.

[12] Keller MJ (2016). A phase 1 randomized placebo-controlled safety and pharmacokinetic trial of a tenofovir disoproxil fumarate vaginal ring. AIDS.

[13] Abdool Karim Q (2010). Effectiveness and safety of tenofovir gel, an antiretroviral microbicide, for the prevention of HIV infection in women. Science.

[14] Grant RM (2010). Preexposure chemoprophylaxis for HIV prevention in men who have sex with men. N. Engl. J. Med..

[15] Baeten JM (2012). Antiretroviral prophylaxis for HIV prevention in heterosexual men and women. N. Engl. J. Med..

[16] Thigpen MC (2012). Antiretroviral preexposure prophylaxis for heterosexual HIV transmission in Botswana. N. Engl. J. Med..

[17] Choopanya K (2013). Antiretroviral prophylaxis for HIV infection in injecting drug users in Bangkok, Thailand (the Bangkok Tenofovir Study): A Randomised, double-blind, placebo-controlled phase 3 trial. Lancet.

[18] Molina JM (2015). On-demand preexposure prophylaxis in men at high risk for HIV-1 infection. N. Engl. J. Med..

[19] Marcus JL (2016). Preexposure prophylaxis for HIV prevention in a large integrated health care system: Adherence, renal safety, and discontinuation. J. Acquir. Immune Defic. Syndr..

[20] McCormack S (2016). Pre-exposure prophylaxis to prevent the acquisition of HIV-1 infection (PROUD): Effectiveness results from the pilot phase of a pragmatic open-label randomised trial. Lancet.

[21] Hare, C. B. *et al.* in *2019 Conference on Retroviruses and Opportunistic Infections (CROI).* Abstract Number 104 (CROI, Alexandria, VA).

[22] Muchomba FM, Gearing RE, Simoni JM, El-Bassel N (2012). State of the science of adherence in pre-exposure prophylaxis and microbicide trials. JAIDS.

[23] Amico KR, Mansoor LE, Corneli A, Torjesen K, van der Straten A (2013). Adherence support approaches in biomedical HIV prevention trials: Experiences, insights and future directions from four multisite prevention trials. AIDS Behav..

[24] Gengiah TN, Moosa A, Naidoo A, Mansoor LE (2014). Adherence challenges with drugs for pre-exposure prophylaxis to prevent HIV infection. Int. J. Clin. Pharm..

[25] Hendrix, C. W. in *2014 Conference on Retroviruses and Opportunistic Infections (CROI).* Oral Abstract 61 (CROI, Alexandria, VA).

[26] Spreen WR, Margolis DA, Pottage JC (2013). Long-acting Injectable antiretrovirals for HIV treatment and prevention. Curr. Opin. HIV AIDS.

[27] Dolgin E (2014). Long-acting HIV drugs advanced to overcome adherence challenge. Nat. Med..

[28] Landovitz RJ (2021). Cabotegravir for HIV prevention in cisgender men and transgender women. N. Engl. J. Med..

[29] Marzinke MA (2021). Characterization of HIV infection in cisgender men and transgender women who have sex with men receiving injectable cabotegravir for HIV prevention: HPTN 083. J. Infect. Dis..

[30] Durham SH, Chahine EB (2021). Cabotegravir-rilpivirine: The first complete long-acting injectable regimen for the treatment of HIV-1 infection. Ann. Pharmacother..

[31] Gunawardana M (2015). Pharmacokinetics of long-acting tenofovir alafenamide (GS-7340) subdermal implant for HIV prophylaxis. Antimicrob. Agents Chemother..

[32] Gunawardana M (2020). Multispecies evaluation of a long-acting tenofovir alafenamide subdermal implant for HIV prophylaxis. Front. Pharmacol..

[33] Schlesinger E (2016). A Tunable, biodegradable, thin-film polymer device as a long-acting implant delivering tenofovir alafenamide fumarate for hiv pre-exposure prophylaxis. Pharm. Res..

[34] Chua CYX (2018). Transcutaneously refillable nanofluidic implant achieves sustained level of tenofovir diphosphate for HIV pre-exposure prophylaxis. J. Control. Release.

[35] Johnson LM (2019). Characterization of a reservoir-style implant for sustained release of tenofovir alafenamide (TAF) for HIV pre-exposure prophylaxis (PrEP). Pharmaceutics.

[36] Su JT (2019). A Subcutaneous implant of tenofovir alafenamide fumarate causes local inflammation and tissue necrosis in rabbits and macaques. Antimicrob. Agents Chemother..

[37] Simpson SM (2020). Design of a drug-eluting subcutaneous implant of the antiretroviral tenofovir alafenamide fumarate. Pharm. Res..

[38] Moss J (2018). *In vitro-in vivo* and *in vivo-in vivo* correlations of TAF release from a novel subdermal implant. AIDS Res. Hum. Retrovir..

[39] Gunawardana M (2018). Multispecies, in vivo evaluation of subdermal implants delivering tenofovir alafenamide: Of mice, dogs and sheep. AIDS Res. Hum. Retrovir..

[40] Romano JW (2021). Tenofovir alafenamide for HIV prevention: Review of the proceedings from the gates foundation long-acting TAF product development meeting. AIDS Res. Hum. Retroviruses.

[41] Parsons TL, Gwenden KN, Marzinke MA (2020). Interspecies differences in tenofovir alafenamide fumarate stability in plasma. Antimicrob. Agents Chemother..

[42] Chapman EH, Kurec AS, Davey FR (1981). Cell volumes of normal and malignant mononuclear cells. J. Clin. Pathol..

[43] Ray AS, Fordyce MW, Hitchcock MJM (2016). Tenofovir alafenamide: A novel prodrug of tenofovir for the treatment of human immunodeficiency virus. Antiviral Res..

[44] Duwal S, Schütte C, von Kleist M (2012). Pharmacokinetics and pharmacodynamics of the reverse transcriptase inhibitor tenofovir and prophylactic efficacy against HIV-1 infection. PLoS ONE.

[45] Prathipati PK, Mandal S, Pon G, Vivekanandan R, Destache CJ (2017). Pharmacokinetic and tissue distribution profile of long acting tenofovir alafenamide and elvitegravir loaded nanoparticles in humanized mice model. Pharm. Res..

[46] Louissaint NA (2013). Single dose pharmacokinetics of oral tenofovir in plasma, peripheral blood mononuclear cells, colonic tissue, and vaginal tissue. AIDS Res. Hum. Retroviruses.

[47] Angel PM, Caprioli RM (2013). Matrix-assisted laser desorption ionization imaging mass spectrometry: *In Situ* molecular mapping. Biochemistry.

[48] Norris JL, Caprioli RM (2013). Analysis of tissue specimens by matrix-assisted laser desorption/ionization imaging mass spectrometry in biological and clinical research. Chem. Rev..

[49] Gessel MM, Norris JL, Caprioli RM (2014). MALDI imaging mass spectrometry: Spatial molecular analysis to enable a new age of discovery. J. Proteomics.

[50] Chumbley CW (2016). Absolute quantitative MALDI imaging mass spectrometry: A case of rifampicin in liver tissues. Anal. Chem..

[51] Birkus G (2007). Cathepsin A is the major hydrolase catalyzing the intracellular hydrolysis of the antiretroviral nucleotide phosphonoamidate prodrugs GS-7340 and GS-9131. Antimicrob. Agents Chemother..

[52] Birkus G (2008). Activation of 9-[(R)-2-[[(S)-[[(S)-1-(Isopropoxycarbonyl)ethyl]amino]phenoxyphosphinyl]-methoxy]propyl]adenine (GS-7340) and other tenofovir phosphonoamidate prodrugs by human proteases. Mol. Pharmacol..

[53] Birkus G (2016). Intracellular activation of tenofovir alafenamide and the effect of viral and host protease inhibitors. Antimicrob. Agents Chemother..

[54] Denton PW (2010). Systemic administration of antiretrovirals prior to exposure prevents rectal and intravenous HIV-1 transmission in humanized BLT mice. PLoS ONE.

[55] Denton PW, Garcia JV (2012). Mucosal HIV-1 transmission and prevention strategies in BLT humanized mice. Trends Microbiol..

[56] Gallay PA (2017). Prevention of vaginal and rectal HIV transmission by antiretroviral combinations in humanized mice. PLoS ONE.

[57] Gallay PA (2018). Protection efficacy of C5A against vaginal and rectal HIV challenges in humanized mice. Open Virol. J..

[58] Baum MM (2020). Highly synergistic drug combination prevents vaginal HIV infection in humanized mice. Sci. Rep..

[59] Chou TC, Talalay P (1984). Quantitative analysis of dose-effect relationships: The combined effects of multiple drugs or enzyme inhibitors. Adv. Enzyme Regul..

[60] Babusis D, Phan TK, Lee WA, Watkins WJ, Ray AS (2013). Mechanism for effective lymphoid cell and tissue loading following oral administration of nucleotide prodrug GS-7340. Mol. Pharmaceut..

[61] Li L, Johnson LM, Krovi SA, Demkovich ZR, van der Straten A (2020). Performance and stability of tenofovir alafenamide formulations within subcutaneous biodegradable implants for HIV Pre-exposure prophylaxis (PrEP). Pharmaceutics.

[62] Pons-Faudoa FP (2020). Viral load reduction in SHIV-positive nonhuman primates *via* long-acting subcutaneous tenofovir alafenamide fumarate release from a nanofluidic implant. Pharmaceutics.

[63] Gengiah, T. *et al.* CAPRISA 018: A phase I/II trial to assess the safety, acceptability, tolerability and pharmacokinetics of a sustained-release tenofovir alafenamide sub-dermal implant for HIV prevention in women.* BMJ Open*, **12**(1), e052880. 10.1136/bmjopen-2021-052880 (2022). 10.1136/bmjopen-2021-052880 PMC8739430 34992111

[64] Anderson PL (2012). Emtricitabine-tenofovir concentrations and pre-exposure prophylaxis efficacy in men who have sex with men. Sci. Transl. Med..

[65] Ward KW (1999). Preclinical pharmacokinetics and interspecies scaling of a novel vitronectin receptor antagonist. Drug. Metab. Dispos..

[66] Ward KW, Azzarano LM, Evans CA, Smith BR (2004). Apparent absolute oral bioavailability in excess of 100% for a vitronectin receptor antagonist (SB-265123) in Rat. I. Investigation of potential experimental and mechanistic explanations. Xenobiotica.

[67] Ward KW, Hardy LB, Kehler JR, Azzarano LM, Smith BR (2004). Apparent absolute oral bioavailability in excess of 100% for a vitronectin receptor antagonist (SB-265123) in Rat. II. Studies implicating transporter-mediated intestinal secretion. Xenobiotica.

[68] Bam RA, Yant SR, Cihlar T (2014). Tenofovir alafenamide is not a substrate for renal organic anion transporters (OATs) and does not exhibit OAT-dependent cytotoxicity. Antivir. Ther..

[69] Robbins BL, Wilcox CK, Fridland A, Rodman JH (2003). Metabolism of tenofovir and didanosine in quiescent or stimulated human peripheral blood mononuclear cells. Pharmacotherapy.

[70] Thompson CG (2015). Mass spectrometry imaging reveals heterogeneous efavirenz distribution within putative HIV reservoirs. Antimicrob. Agents Chemother..

[71] Rosen EP (2016). Analysis of antiretrovirals in single hair strands for evaluation of drug adherence with infrared-matrix-assisted laser desorption electrospray ionization mass spectrometry imaging. Anal. Chem..

[72] Seneviratne HK, Hendrix CW, Fuchs EJ, Bumpus NN (2018). MALDI mass spectrometry imaging reveals heterogeneous distribution of tenofovir and tenofovir diphosphate in colorectal tissue of subjects receiving a tenofovir-containing enema. J. Pharmacol. Exp. Ther..

[73] Thompson CG (2019). Heterogeneous antiretroviral drug distribution and HIV/SHIV detection in the gut of three species. Sci. Transl. Med..

[74] Seneviratne HK, Hamlin AN, Heck CJS, Bumpus NN (2020). Spatial distribution profiles of emtricitabine, tenofovir, efavirenz, and rilpivirine in murine tissues following *in vivo* dosing correlate with their safety profiles in humans. ACS Pharmacol. Transl. Sci..

[75] Kastellorizios M, Tipnis N, Burgess DJ, Lambris JD, Ekdahl KN, Ricklin D, Nilsson B (2015). Immune Responses to Biosurfaces: Mechanisms and Therapeutic Interventions. Advances in Experimental Medicine and Biology.

[76] Vegas AJ (2016). Combinatorial hydrogel library enables identification of materials that mitigate the foreign body response in primates. Nat. Biotechnol..

[77] Kuo, S.- H. & Kuzma, P. Long Term Drug Delivery Devices with Polyurethane Based Polymers and their Manufacture. USA patent U.S. Patent 7,842,303 B2 (2010).

[78] Gunawardana M, Baum MM, Smith TJ, Moss JA (2014). An intravaginal ring for the sustained delivery of antibodies. J. Pharm. Sci..

[79] National Research Council. *Guide for the Care and Use of Laboratory Animals* 8th edn, Vol. 220 (The National Academies Press, 2001).

[80] ISO. ISO 10993-6: Biological Evaluation of Medical Devices—Part 6: Tests for Local Effects After Implantation. 29 (International Organization for Standardization, Geneva, Switzerland, 2016).

[81] Hummert P, Parsons TL, Ensign LM, Hoang T, Marzinke MA (2018). Validation and implementation of liquid chromatographic-mass spectrometric (LC-MS) methods for the quantification of tenofovir prodrugs. J. Pharm. Biomed. Anal..

