# Supplementary material for: Fundamental aspects of long-acting tenofovir alafenamide delivery from subdermal implants for HIV prophylaxis
Source: Sci Rep. 2022 May 17;12:8224. doi: 10.1038/s41598-022-11020-2 (PMC9114338; doi:10.1038/s41598-022-11020-2)
Supplement: Supplementary file 1 — Supplementary Information. [file 41598_2022_11020_MOESM1_ESM.docx]

Fundamental Aspects of Long-acting Tenofovir Alafenamide Delivery from Subdermal Implants for HIV Prophylaxis

Manjula Gunawardana^1^, Mariana Remedios-Chan^1^, Debbie Sanchez^1^, Simon Webster^1^, Amalia E. Castonguay^1^, Paul Webster^1^, Christopher Buser^1^, John A. Moss^1^, MyMy Trinh^2^, Martin Beliveau^2^, Craig W. Hendrix^3^, Mark A. Marzinke^3,4^, Michael Tuck^5^, Richard M. Caprioli^5^, Michelle L. Reyzer^5^, Joseph Kuo^6^, Philippe A. Gallay^6^, Marc M. Baum^1*^

^1^Department of Chemistry, Oak Crest Institute of Science, 128-132 W. Chestnut Ave., Monrovia, CA, USA

^2^Certara Integrated Drug Development, 2000 Peel Street, Suite 570, Montreal, Quebec, Canada

^3^Department of Medicine, Johns Hopkins University, 600 N. Wolfe Street, Baltimore, MD, USA

^4^Department of Pathology, Johns Hopkins University, 600 N. Wolfe Street/Carnegie 417, Baltimore, MD, USA

^5^Department of Biochemistry, Vanderbilt University, 9160 MRB III, 465 21st Ave. South, Nashville, TN, USA

^6^Department of Immunology & Microbiology, The Scripps Research Institute, 10550 North Torrey Pines Road, La Jolla, CA, USA

^*^email: m.baum@oak-crest.org

**Supplementary Information**

| **Supplementary Table 1. Summary of MALDI IMS conditions used in the analysis of flash-frozen dermal tissue samples collected adjacent to TAF implants.** | | | | |
| --- | --- | --- | --- | --- |
| **Analyte** | **Polarity** | **Precursor Ion Identity** | **Precursor Ion Mass** | **Fragment Ions** |
| TAF | Positive | [M+H]^+^ | 477 | **270, 346** |
| Metabolite X | Positive | [M+H]^+^ | 359 | **270, 288** |
| Metabolite Y | Positive | [M+H]^+^ | 401 | **270** |
| TFV | Negative | [M-H]^-^ | 286 | 111, **134**, **151** |
| TFV-MP | Negative | [M-H]^-^ | 366 | **268, 348** |
| TFV-DP | Negative | [M-H]^-^ | 446 | **159, 348** |
